# Further evidence supporting a potential role for ADH1B in obesity

**DOI:** 10.1038/s41598-020-80563-z

**Published:** 2021-01-21

**Authors:** Liza D. Morales, Douglas T. Cromack, Devjit Tripathy, Marcel Fourcaudot, Satish Kumar, Joanne E. Curran, Melanie Carless, Harald H. H. Göring, Shirley L. Hu, Juan Carlos Lopez-Alvarenga, Kristina M. Garske, Päivi Pajukanta, Kerrin S. Small, Craig A. Glastonbury, Swapan K. Das, Carl Langefeld, Robert L. Hanson, Wen-Chi Hsueh, Luke Norton, Rector Arya, Srinivas Mummidi, John Blangero, Ralph A. DeFronzo, Ravindranath Duggirala, Christopher P. Jenkinson

**Affiliations:** 1 South Texas Diabetes and Obesity Institute Department of Human Genetics, School of Medicine, University of Texas Rio Grande Valley, Edinburg/Harlingen/Brownsville, TX USA; 2 South Texas Veterans Health Care System, San Antonio, TX USA; 3 Department of Medicine, University of Texas Health San Antonio, San Antonio, TX USA; 4 Department of Population Health, Texas Biomedical Research Institute, San Antonio, TX USA; 5 University of Texas Health Houston, School of Public Health, Brownsville, TX USA; 6 Department of Human Genetics, David Geffen School of Medicine at UCLA, Los Angeles, CA USA; 7 King’s College London, London, UK; 8 Internal Medicine-Endocrinology and Metabolism, Wake Forest School of Medicine, Winston-Salem, NC USA; 9 Department of Biostatistics and Data Science, Wake Forest School of Medicine, Winston-Salem, NC USA; 10 Phoenix Epidemiology and Clinical Research Branch, NIDDK, Phoenix, AZ USA

**Keywords:** Mechanisms of disease, Gene expression, Gene regulation, Medical genetics, Transcriptomics

## Abstract

Insulin is an essential hormone that regulates glucose homeostasis and metabolism. Insulin resistance (IR) arises when tissues fail to respond to insulin, and it leads to serious health problems including Type 2 Diabetes (T2D). Obesity is a major contributor to the development of IR and T2D. We previously showed that gene expression of alcohol dehydrogenase 1B (ADH1B) was inversely correlated with obesity and IR in subcutaneous adipose tissue of Mexican Americans. In the current study, a meta-analysis of the relationship between ADH1B expression and BMI in Mexican Americans, African Americans, Europeans, and Pima Indians verified that BMI was increased with decreased ADH1B expression. Using established human subcutaneous pre-adipocyte cell lines derived from lean (BMI < 30 kg m^−2^) or obese (BMI ≥ 30 kg m^−2^) donors, we found that ADH1B protein expression increased substantially during differentiation, and overexpression of ADH1B inhibited fatty acid binding protein expression. Mature adipocytes from lean donors expressed ADH1B at higher levels than obese donors. Insulin further induced ADH1B protein expression as well as enzyme activity. Knockdown of ADH1B expression decreased insulin-stimulated glucose uptake. Our findings suggest that ADH1B is involved in the proper development and metabolic activity of adipose tissues and this function is suppressed by obesity.

## Introduction

The twin epidemics of obesity and type 2 diabetes (T2D), present major economic, social and medical challenges^1–3^. Both metabolic disorders represent insulin resistant states. We and others have demonstrated that T2D is an inherited disease characterized by insulin resistance in insulin target tissues, notably adipose tissue, skeletal muscle, liver and pancreatic b-cells^4–6^. Obesity, the excess accumulation of fat mass, is a pervasive form of insulin resistance and a major risk factor for T2D^7^. Insulin resistance typically occurs within a cluster of highly correlated traits including obesity, T2D, hypertension and dyslipidemia. This cluster of traits, *aka* the Metabolic Syndrome, is a predictor of cardiovascular disease and stroke. The prevalence rates of obesity and T2D are particularly high in US ethnic minorities including Mexican Americans, an ethnic group with a three to fourfold higher incidence of T2D than Europeans^8,9^ and we have published evidence of alarming levels of obesity, metabolic syndrome, prediabetes and related disorders in Mexican American offspring of diabetic parents^10^.

We previously showed that obesity, insulin resistance and T2D traits have a strong genetic basis and high prevalence rates in Mexican Americans^11–13^. However, the molecular mechanisms underlying obesity, insulin resistance and T2D remain unclear. They are complex multifactorial metabolic disorders with a heterogeneous contribution from multiple genes and environmental factors as well as the complex interactions between these factors^14–18^. Ongoing efforts seek to localize and characterize obesity and T2D susceptibility genes using a variety of molecular approaches: genome-wide linkage, genome-wide association studies (GWAS), whole genome sequencing and genome-wide gene expression (transcriptomics) studies including the approach described here. GWAS is a widely used study design with several notable successes in localizing putative obesity and T2D susceptibility genes/variants. However, to date, in most cases the identity of the causal genes and functional relevance of the implicated genetic variants remains to be determined. Of further concern, common variants identified by GWAS explain only ~ 10% of the *total* trait variance for T2D and ~ 5% of the variance for obesity, indicating that a large proportion of the heritability (genetic effect) remains unexplained^19–21^. Thus, there are serious gaps in our understanding of the molecular basis of obesity.

We previously reported^22^ that gene expression of the cytosolic enzyme, alcohol dehydrogenase 1B (ADH1B) was strongly and significantly inversely correlated with 15 obesity-related traits tested. We made this discovery in our ongoing population genetic studies of T2D, obesity and insulin resistance in a Mexican American population from San Antonio, Texas. Those studies consisted of genome-wide gene expression analyses in abdominal subcutaneous adipose tissue following a standard 75 g glucose Oral Glucose Tolerance Test in 75 subjects. Unexpectedly, *ADH1B* in fasting adipose tissue emerged as the strongest causal candidate gene for obesity/insulin resistance (OB/IR). In concordance with these results, adipose ADH1B protein expression was also significantly increased fivefold in low BMI (N = 6, BMI = 27.5 ± 2.5 kg m^−2^) individuals versus high BMI (N = 6, BMI = 42.4 ± 7.3 kg m^−2^) individuals as shown by Western blot analysis^22^.

The effect of distant regulatory regions on *ADH1B* is known to be mediated by three-dimensional chromatin conformational changes leading to close contact between proteins bound to regulatory and promoter regions. In subcutaneous adipose tissue we identified a tight cluster of 20 eQTLs approximately 56 kb upstream of the *ADH1B* gene which are associated with expression. This cluster is specific to subcutaneous adipose tissue and no similar cluster is observed in visceral adipose tissue, mammary gland or liver. The *ADH1B* regulatory eQTLs interact with the promoter through chromatin conformational contact. There is also an ATACseq double peak immediately upstream of the transcriptional start site in adipose tissue (UCSC browser, accessed 3/23/2019), indicating potential open “active” chromatin available for the binding of transcription factors.

Regarding overall regulation of the *ADH1B* gene, we recently published^23^ findings using promoter capture HiC (pCHi-C) in human adipose tissue and using adipose RNA-seq data found that adipose expression of *ADH1B* was the most strongly BMI-correlated gene among the top 42 genes involved in chromosome looping interactions with their cis-eQTL SNPs (METSIM study linear regression p = 10^–20^; TwinsUK p = 10^–71^). The results showed strong single gene effect sizes and the same negative correlations we previously observed (β − 0.21 to − 0.58). This result highlights the strong regulation of *ADH1B* by cis-regulated chromosomal promoter interaction elements in adipocytes and emphasizes the need for further exploration of the role of eQTLs in the *ADH1B* upstream region in its expression.

Despite the high and specific expression of *ADH1B* in adipose tissue, the functional role of ADH1B protein in adipose and its relationship to obesity is unknown. In the current study, we performed molecular studies, using human subcutaneous pre-adipocyte cell lines to examine the role of ADH1B protein in adipocytes and the potential involvement of ADH1B in the development of obesity. We found ADH1B protein expression regulates the adipokine fatty acid binding protein 4 (FABP4, also known as adipocyte FABP or aP2) during adipocyte differentiation and ADH1B expression was suppressed by OB/IR. Moreover, our results demonstrated that ADH1B may play a functional role in insulin action: (1) insulin treatment resulted in a substantial increase in ADH1B protein expression and enzymatic activity; (2) inhibition of AKT phosphorylation, suppressed ADH1B expression, indicating that insulin promotes ADH1B expression through an AKT-dependent pathway; and (3) knockdown of ADH1B decreased insulin-mediated glucose uptake in adipocytes. These preliminary findings point to an unsuspected potential role for ADH1B in adipocytes and the adipocyte response to insulin, which may be a factor in obesity and related metabolic disorders.

## Results

### Low *ADH1B* expression is a universal characteristic concomitant with obesity

**Figure 1:**
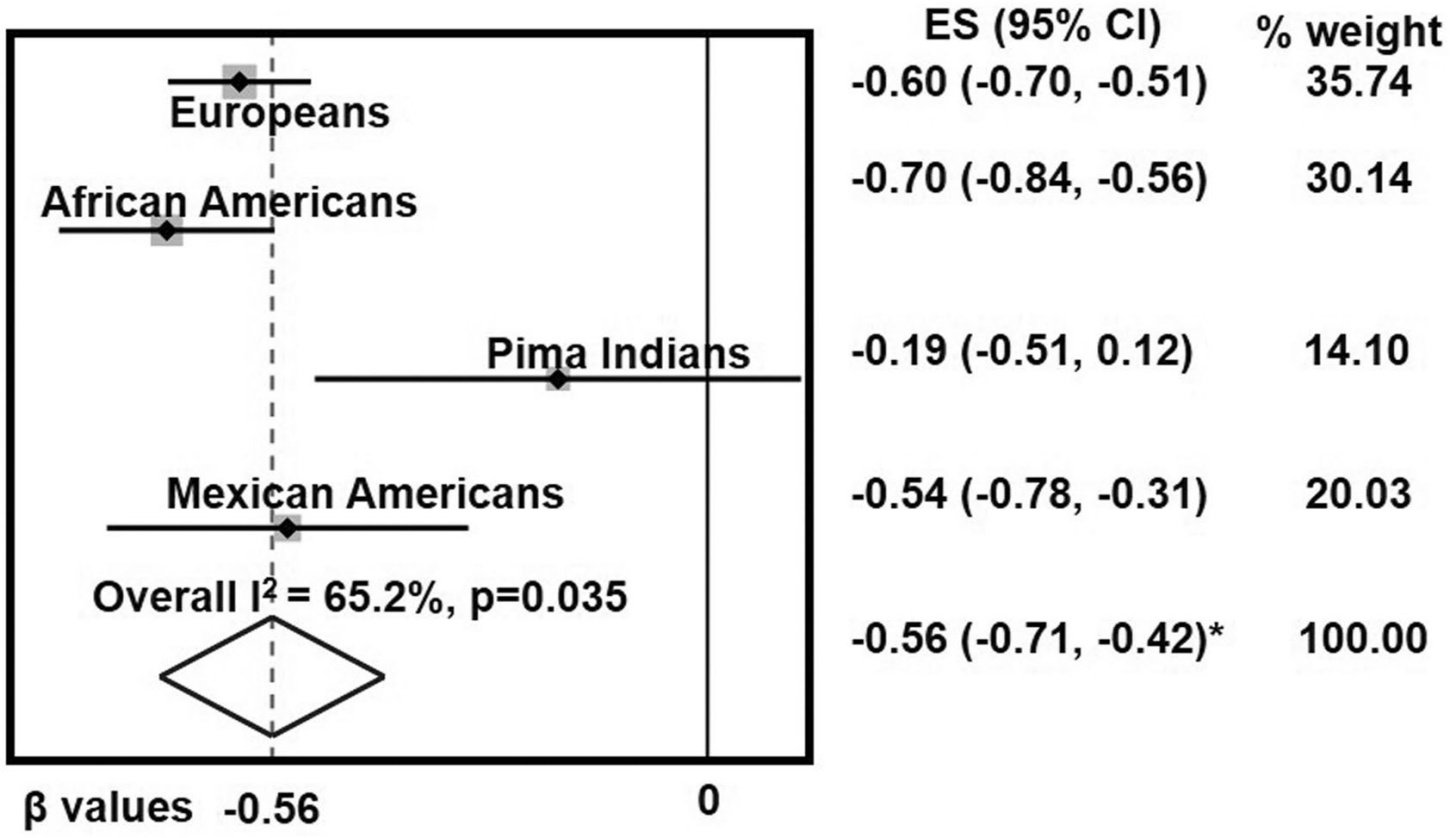
Global relevance of the inverse association of ADH1B expression with obesity traits. Meta-analysis, using a random effects model, was performed to evaluate the relationship between adipose ADH1B mRNA expression and BMI in populations from four ethnic groups (Europeans, African Americans, Pima Indians, and Mexican Americans) as indicated. The data show point estimates and 95% confidence intervals for BMI β-values in each ethnic group. Gray squares represent weighted contributions from each study, and the diamond represents the overall summary statistic. For each standard deviation of decreased *ADH1B* expression, BMI was increased by 0.56 standard deviations, *P < 0.001.

Given our previous results in Mexican Americans, we performed an aggregation of standardized beta values from correlations (using meta-analysis tools) between *ADH1B* expression and BMI or fasting plasma glucose, respectively, in four populations. We collected BMI and glucose data from: Europeans, African Americans, Pima Indians and Mexican Americans. A random effects model was used due to the heterogeneity of the studies (I^2^ = 65.2% for BMI and 86.1% for glucose). These analyses were performed with STATA 15.1 (StataCorp LLC, TX). As depicted in Fig. 1, the meta-analysis for BMI showed a pooled standardized beta of − 0.56 (95% CI − 0.71, − 0.42; p < 0.001) and for fasting plasma glucose it was − 0.17 (95%CI − 0.38, 0.04; p = 0.103).

This analysis supports a strong effect for BMI with *ADH1B* expression across four populations, and no effect for glucose. *ADH1B* expression increased for each decrement of 0.56 standard deviations in BMI. The BMI effect was most pronounced in African Americans, followed by Europeans and Mexican Americans and was suggestive in Pima Indians (Fig. 1). These findings highlight the global relevance of the relationship between *ADH1B* expression and obesity. The finding in Mexican Americans is interesting, given that this is an admixed population deriving its ancestral origins from both European and Amerindian populations and the result for Mexican Americans appears to fall between the results for the cohorts of these populations.

### *ADH1B* is highly and specifically expressed in subcutaneous adipose tissue

According to the most recently available data from the Genotype-Tissue Expression (GTEx) portal^22^ (https://gtexportal.org, accessed 4/3/2019), *ADH1B* represents the most highly expressed gene in human subcutaneous adipose tissue, at a level of 1300 transcripts per million (TPM), or 100%, in comparison to 52 other tissues examined, including visceral adipose tissue (73% of *ADH1B* expression levels), breast tissue (58%), and liver (58%). It is substantially more highly expressed than its isoforms *ADH1A* and *ADH1C*, which are almost exclusively expressed in liver. The expression levels of both were at 21% of the level of *ADH1B*. This result agrees with our previous findings^22^.

### Expression of ADH1B protein was upregulated during adipogenesis

**Figure 2:**
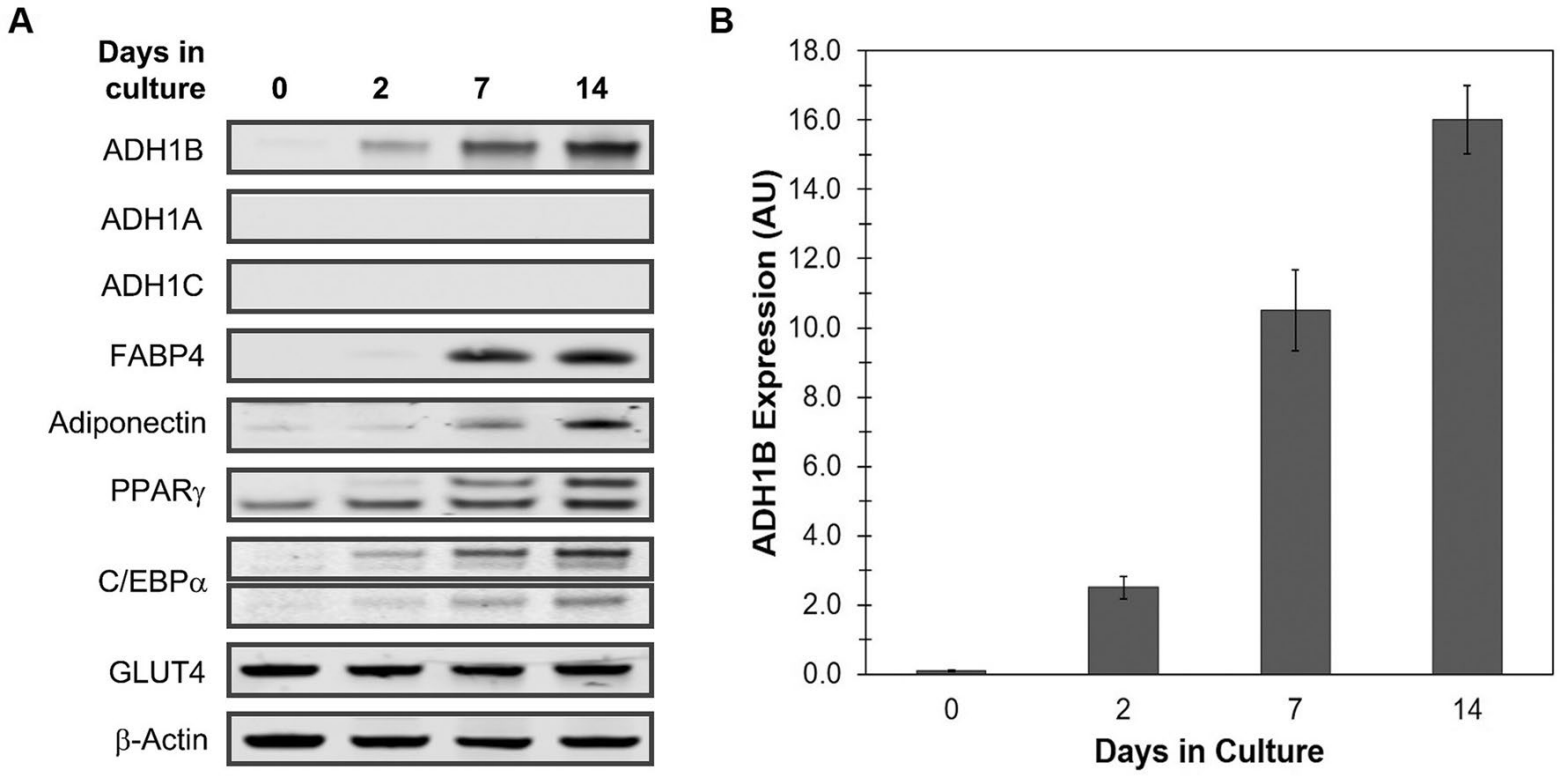
Expression levels of ADH1 in differentiating human subcutaneous pre-adipocytes. (**A**) Representative composite image of immunoblot analysis. Total protein was isolated at the indicated time points during differentiation of human subcutaneous pre-adipocytes (Day 0) to cultured mature adipocytes (Day 14), resolved by SDS-PAGE, and immunoblotted with antibodies specific for the three ADH1 isoforms (ADH1A, ADH1B, ADH1C); the adipokines FABP4, adiponectin, PPARγ, and C/EBPα; and the adipose-specific glucose transporter GLUT4. β-Actin was used as a loading control. Full length blots are presented in Supplementary Figures S7 and S13. (**B**) Quantitative analysis of ADH1B protein expression. Western blot analysis was performed at least three times. Fluorescent signal intensity was quantified and normalized to β-Actin control. Data is presented as the mean ± s.e.m. AU, Arbitrary Units.

To investigate the potential function of ADH1B protein in adipose, we first examined in vitro protein expression levels of ADH1B in differentiated subcutaneous human pre-adipocytes derived from adipose tissue. Pre-adipocytes (Day 0) were differentiated to mature adipocytes (Day 14) in culture, as shown by changes in cell morphology and positive-staining of intracellular lipid droplets with Oil Red O lipophilic dye (Supplementary Fig. S1A). Additionally, Western blot analysis of total protein isolated at Days 0, 2, 7, and 14 during differentiation, shown in Fig. 2A, confirmed increased expression levels of the known differentiation markers fatty acid binding protein 4 (FABP4, also known as adipocyte FABP or aP2), adiponectin (also known as AdipoQ), peroxisome proliferator-activated receptor gamma (PPARγ) and CCAAT/enhancer-binding protein alpha (C/EBPα). GLUT4, an insulin-regulated glucose transporter primarily located in adipose tissue and striated muscle, was constitutively expressed in preadipocytes and adipocytes (Fig. 2A).

Quantification of Western blot analysis replicates showed in vitro ADH1B protein expression levels increased ~ 170-fold from Day 0 to Day 14 (Fig. 2A,B). ADH1B expression levels appeared to plateau and remained relatively unchanged once complete differentiation had been attained (Supplementary Fig. S2). Moreover, cultured mature adipocytes from three different donors with similar age, gender and BMI showed relatively comparable expression levels of ADH1B during adipogenesis (Supplementary Table S1, Supplementary Fig. S3). Our results also showed that the isoforms ADH1A and ADH1C were not detected by Western blot analysis in either pre-adipocytes or adipocytes, thus densitometry was omitted (Fig. 2A,B).

Interestingly, measurement of mRNA expression levels by quantitative real-time PCR (qPCR) during differentiation revealed mRNA expression levels of *ADH1C*, which is transcribed prior to *ADH1B*, increased from Day 0 to Day 2 but remained at relatively similar levels for the remainder of differentiation (Supplementary Fig. S4A,B). *ADH1B* mRNA was expressed in pre-adipocytes and increased over 100-fold by Day 7 of differentiation; however mRNA expression levels decreased once differentiation was complete at Day 14 (Supplementary Fig. S4B,C). In contrast, mRNA expression of *ADH1A*, which is transcribed following *ADH1B*, was not detected in pre-adipocytes, however it was expressed at relatively similar levels in Days 2 through 14 (Supplementary Fig. S4A,B). These results demonstrate that *ADH1B* is upregulated during adipogenesis for protein expression and there is a molecular mechanism that suppresses transcription once adipogenesis is close to completion, whereas *ADH1A *and *ADH1C* mRNA transcripts appear to be unstable and/or are not translated to protein in adipocytes.

**Figure 3:**
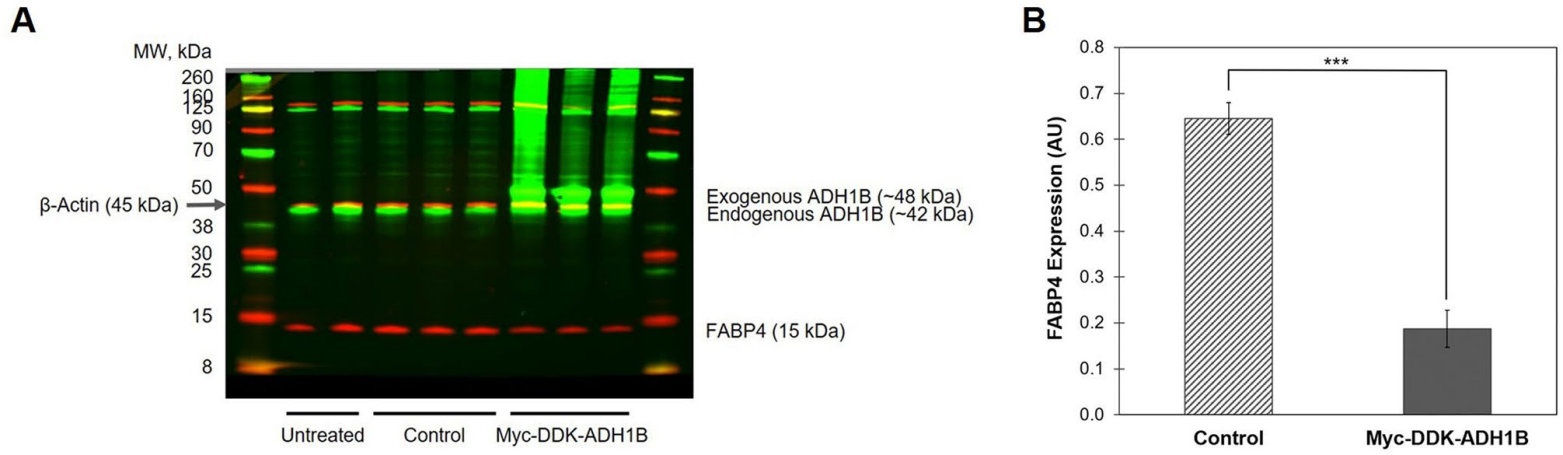
ADH1B regulates FABP4 expression during adipocyte differentiation. (**A**) Immunoblot analysis of cell lysates from adipocytes following differentiation and transduction with lentiviruses. Pre-adipocytes were transduced with lentivirus containing empty vector (Control) or vector encoding myc-DDK-tagged human ADH1B on Day 0 and Day 10 of differentiation in triplicate. Untreated pre-adipocytes were differentiated in duplicate and utilized as an additional control. Total protein was collected following complete differentiation, resolved by SDS-PAGE, and immunoblotted with antibodies specific for ADH1B, FABP4, and β-actin control. (**B**) Quantitative analysis of FABP4 expression in cells overexpressing ADH1B compared to empty vector control. Biological replicates were processed on the same Western blot. Fluorescence intensity was quantified and normalized to β-Actin control. Data is presented as the mean ± s.e.m. AU, Arbitrary Units; ***P = 0.0005.

Our findings imply that ADH1B gene and protein expression is well-regulated during differentiation and therefore ADH1B may play an important role in adipogenesis. In fact, a study by Kerr et al.^31^ demonstrated siRNA-knockdown of ADH1B in pre-adipocytes reduced adipogenesis as evidenced by a decrease in lipid accumulation. To further test whether ADH1B is needed for differentiation, we utilized a lentiviral vector system to overexpress ADH1B protein in differentiating human subcutaneous pre-adipocytes. Untreated cells and cells transduced with lentivirus expressing empty vector (Control) expressed endogenous ADH1B following differentiation as expected, whereas cells transduced with lentivirus expressing myc-DDK-tagged exogenous ADH1B clearly expressed both endogenous ADH1B and exogenous ADH1B following differentiation (Fig. 3A). Western blot analysis with anti-DDK antibody confirmed the presence of myc-DDK-tagged ADH1B (Supplementary Fig. S5A). Interestingly, adipocytes overexpressing ADH1B, showed a significant (P = 0.0005) ~ threefold decrease in FABP4 expression compared to control (Fig. 3A,B), however there were no significant changes in expression levels of C/EBPα or PPARγ (Supplementary Fig. S5B). FABP4 is highly expressed in adipose tissue and is critical to the biological function of adipocytes. The results suggest ADH1B may be involved in the regulation of FABP4 in adipocytes and consequently may also be important to adipogenesis and adipocyte cellular function.

### ADH1B protein expression in adipocytes decreased with increasing BMI

**Figure 4:**
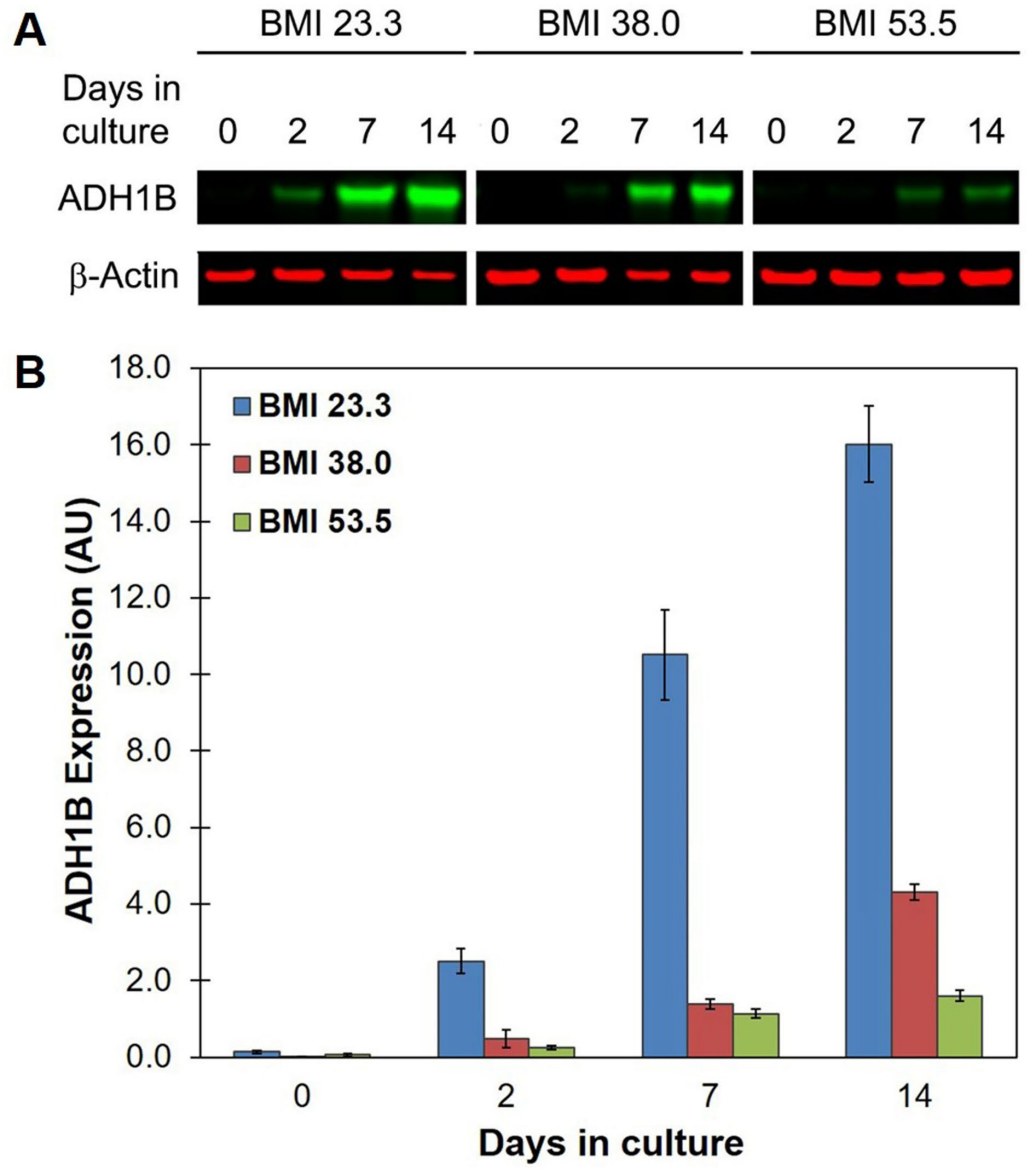
ADH1B protein expression decreased with increasing BMI. (**A**) Representative composite immunoblot image of cell lysates from 3 pre-adipocyte cell lines derived from lean (BMI < 30 kg m^−2^) or obese (BMI ≥ 30 kg m^−2^) individuals during adipogenesis. Total protein was isolated at the indicated time points during differentiation, resolved by SDS-PAGE, and immunoblotted with antibodies specific for ADH1B and β-Actin loading control. Representative full-length blot is presented in Supplementary Figure S14. (**B**) Quantitative analysis of ADH1B expression. Western blot analysis was performed at least four times. Fluorescence intensity was quantified and normalized to β-Actin control. Data is presented as the mean ± s.e.m. AU, Arbitrary Units.

Next, we compared in vitro ADH1B mRNA and protein expression levels in adipocyte cell lines derived from three different donors with a range of BMIs. For the purposes of these studies, we defined lean as a BMI < 30 kg m^−2^ and obese as the standard of a BMI ≥ 30 kg m^−2^. During adipogenesis, all cell lines demonstrated a similar pattern of expression: mRNA expression increased up to Day 7 and then decreased at Day 14 (Supplementary Fig. S6), again implicating the presence of a mechanism that suppresses *ADH1B* expression at the end of differentiation. At Day 7 and Day 14, *ADH1B* mRNA levels appeared to be highest in cells from obese (BMI 53.5 kg m^−2^ and BMI 38.0 kg m^−2^, respectively) donors (Supplementary Fig. S6). On the other hand, ADH1B protein expression levels were considerably higher in cells from a lean (BMI 23.3 kg m^−2^) donor compared to cells from obese (BMI ≥ 30 kg m^−2^) donors throughout adipogenesis (Fig. 4A,B) which is consistent with our previous findings in adipose tissue^22^.

The data suggests obesity blunts ADH1B protein expression; however, it is possible that ADH1B expression is decreased due to a corresponding decrease in differentiation. Therefore, we compared the levels of lipid accumulation in the three cell lines. Quantification of Oil Red O staining showed cells from the lean (BMI 23.3 kg m^−2^) donor and the donor with a BMI of 38.0 kg m^−2^ had similar levels of staining, whereas lipid accumulation in the enlarged, or hypertrophic, adipocytes from the donor with a BMI of 53.5 kg m^−2^ was dramatically reduced (Supplementary Fig. S1B). Comparison of the expression levels of the differentiation markers PPARγ, C/EBPα, and FABP4 in lean and obese (BMI 38.0 kg m^−2^) adipocytes by Western blot analysis confirmed the two cell types underwent similar levels of adipogenesis (Supplementary Fig. S7). Given that the donor with BMI 53.5 kg m^−2^ was diabetic (Supplementary Table S1) and had reduced adipogenesis due to the cells being hypertrophic, we utilized only the cells from the “healthy” obese donor with BMI 38.0 kg m^−2^ for further experimentation.

Insulin resistance (IR) is a hallmark of obesity, therefore, we wished to confirm whether “healthy” obese adipocytes displayed IR in vitro. We treated adipocytes from the lean or obese donor with increasing doses of insulin (0, 0.05, 0.20, 0.50, 1.0 µM) following 12 h starvation. AKT serine/threonine kinase is phosphorylated during insulin signal transduction to activate AKT signaling for glucose uptake and other essential cell signaling mechanisms. Hence, it has become a commonly used indicator of insulin sensitivity. Western blot analysis confirmed that lean adipocytes were insulin sensitive and showed an increase in AKT phosphorylation relative to total AKT expression levels, in a dose-dependent manner (Supplementary Fig. S8). In contrast, obese adipocytes displayed significantly lower levels of AKT activation, particularly at 0.50 and 1.00 µM insulin (P = 0.004 and P = 0.01, respectively), indicating that these cells were comparatively insulin resistant. These findings demonstrate that OB/IR blunts ADH1B protein expression by an unknown mechanism.

### Insulin signaling stimulates ADH1B protein expression in adipocytes via AKT

**Figure 5:**
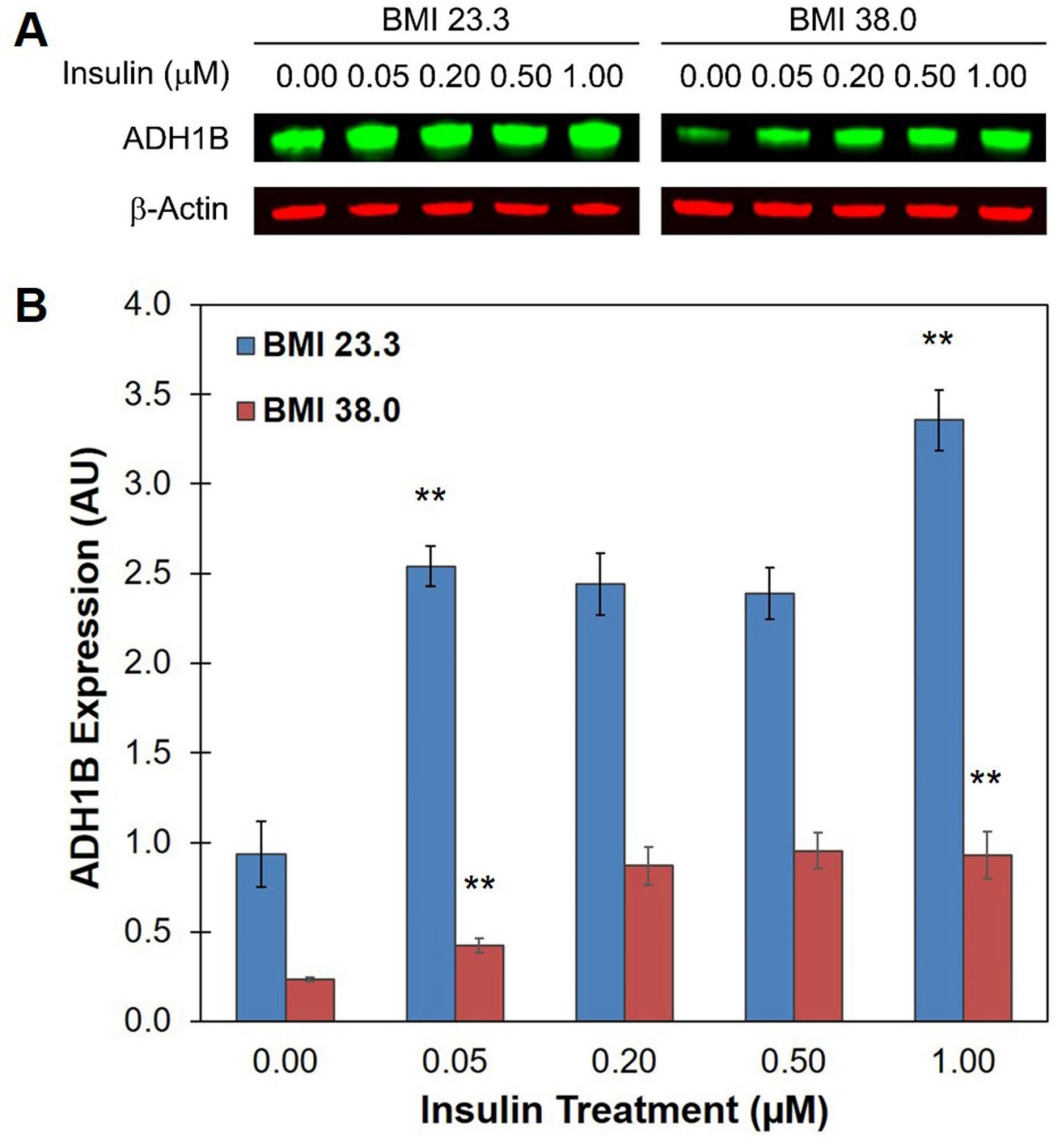
Insulin promotes expression of ADH1B in lean and obese adipocytes. (**A**) Representative composite immunoblot image of cell lysates from lean (BMI < 28 kg m^−2^) and obese (BMI ≥ 30 kg m^−2^) adipocytes following treatment with increasing doses of insulin. Cells were starved for 12 h and then treated with the indicated dose of insulin for 1 h. Total protein was isolated, resolved by SDS-PAGE, and immunoblotted with antibodies specific for ADH1B and β-Actin loading control. Full length blot is presented in Supplementary Figure S14. (**B**) Quantitative analysis of ADH1B expression. Western blot analysis was performed in quadruplicate. Fluorescence intensity was quantified and normalized to β-Actin control. Data is presented as the mean ± s.e.m. AU, Arbitrary Units; **P < 0.001.

We found that insulin treatment stimulated an increase in ADH1B expression in both lean and obese/IR adipocytes (Fig. 5A,B). Pre-adipocytes were differentiated into mature adipocytes, starved and then exposed to increasing doses of insulin for 1 h. Quantification of Western blot analysis replicates revealed that ADH1B expression significantly (P < 0.001) increased in both lean and obese cells following treatment with insulin compared to untreated control (Fig. 5A,B). Treatment with as little as 0.05 µM concentration of insulin resulted in an approximately twofold increase in ADH1B expression in both cell types, whereas treatment with 1.00 µM concentration of insulin yielded an approximately threefold or fourfold increase of ADH1B levels in lean or obese adipocytes, respectively. ADH1B protein expression was upregulated by insulin signaling for up to 3 h following addition of either 0.05 µM or 1.0 µM insulin (Supplementary Fig. S9A,B).

To determine how insulin signaling can induce ADH1B protein expression, cultured mature adipocytes were pretreated with 0.3 µg/ml actinomycin D (ActD), a DNA intercalator that blocks RNA polymerase progression, for 15 min (0.25 h); then 0.05 µM insulin was added and cells were incubated in ActD ± insulin for a total of 4 h (Supplementary Fig. S10). Total RNA was isolated at 0, 0.25, 0.5, 1, 2, and 4 h following ActD treatment and the mRNA levels of *ADH1B* was determined by qPCR*.* The data showed that in adipocytes treated with actinomycin D alone (+ActD), levels of *ADH1B* mRNA relative to untreated control (0 h) remained the same until the 1 h time point (Supplementary Fig. S10). Relative *ADH1B* mRNA levels were significantly reduced 2 h and 4 h (P = 0.0007 and P = 0.001, respectively) after treatment with ActD compared to 1 h, indicating ActD effectively suppressed *ADH1B* transcription in adipocytes following 2 h incubation. Addition of insulin to cells treated with ActD showed a significant (P = 0.0001) decrease in *ADH1B* mRNA levels between the 0.5 h and 1 h time points, however addition of insulin had no effect on mRNA levels compared to ActD alone. The results imply ActD effectively blocked insulin activity and therefore insulin signaling regulates *ADH1B* expression at the transcriptional level.

Next, we treated cultured adipocytes with 0.178 mM cycloheximide, a fungicide known to block the translation step in protein synthesis, for 1 h prior to treatment with 0.05 µM insulin for 3 h. Western blot analysis results showed that adipocytes treated with both cycloheximide and insulin express ADH1B protein at a level similar to untreated control and at a level much less than cells treated with insulin alone, indicating that cycloheximide effectively blocked the increase in ADH1B expression induced by insulin (Supplementary Fig. S11). Western blot analysis also showed AKT activation by insulin signaling, confirming that cycloheximide alone was responsible for the decrease in ADH1B expression. The data demonstrated that insulin signaling regulates ADH1B expression at the translational level as well.

**Figure 6:**
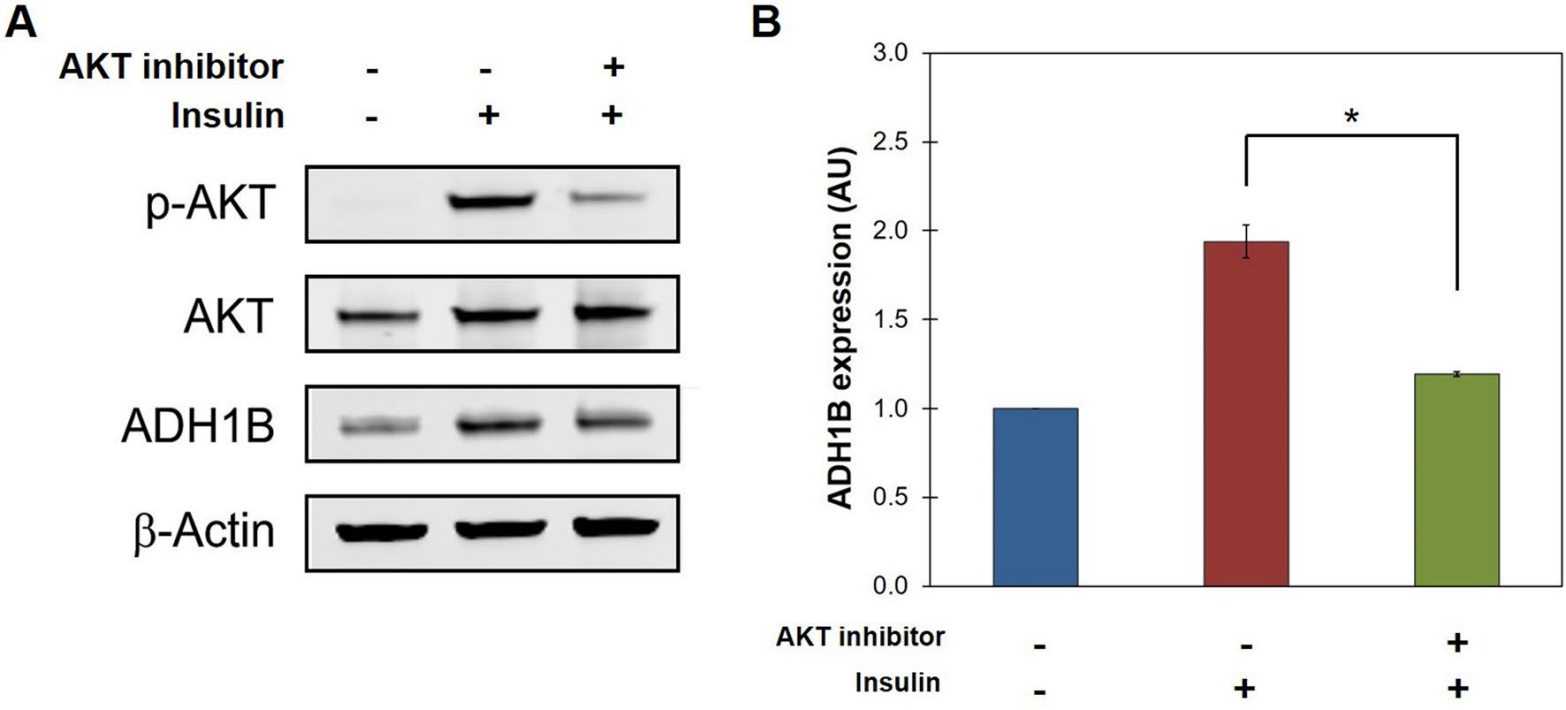
AKT is involved in insulin-mediated ADH1B expression. (**A**) Representative composite image of ADH1B expression with ( +) and without (−) treatment with AKT inhibitor and/or insulin. Following differentiation, cultured adipocytes were starved for 12 h and then treated with 20 µM AKT1/2-specific inhibitor (+ AKT inhibitor) for 1 h prior to treatment with 1.0 µM insulin (+ Insulin) for 1 h. Non-treated cells (−Insulin, −AKT inhibitor) were utilized as control. Total protein was isolated, resolved by SDS-PAGE, and immunoblotted with antibodies specific for phosphorylated AKT (p-AKT) or total AKT and ADH1B. β-Actin was used as loading control. A representative full-length blot is presented in Supplementary Figure S16. (**B**) Quantitative analysis of ADH1B expression relative to non-treated control. Western blot analysis was performed in triplicate. Fluorescence intensity was quantified and normalized to β-Actin control. Data is presented as the mean ± s.e.m. AU, Arbitrary Units; *P < 0.01.

Finally, mature adipocytes were treated with an AKT-specific inhibitor to suppress phosphorylation of AKT to the active form. Western blot analysis of expression levels of total AKT protein (AKT), phosphorylated AKT (p-AKT), and ADH1B revealed that following treatment with 0.05 µM insulin for 1 h, AKT was phosphorylated and ADH1B expression increased in comparison to no treatment control (Fig. 6A,B). However, quantification of Western blot analysis demonstrated that treatment with 20 µM AKT-specific inhibitor 1 h prior to the addition of insulin significantly (P < 0.01) suppressed the increase in ADH1B expression observed in cells treated with insulin alone (Fig. 6B). Thus, insulin stimulated ADH1B expression required AKT phosphorylation, implying AKT kinase is an upstream regulator of ADH1B in the insulin signaling pathway.

### ADH1B plays a functional role in the cell response to insulin

**Figure 7:**
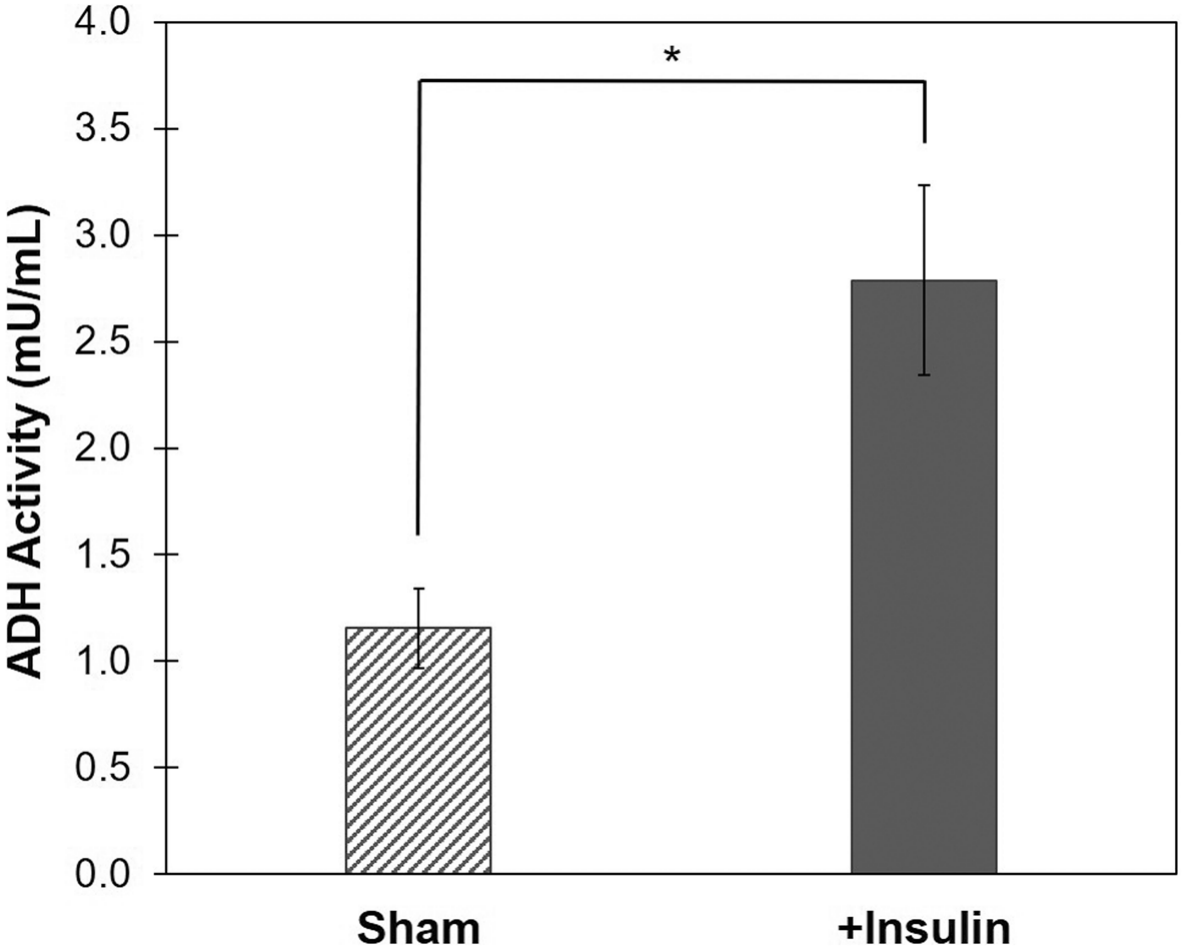
Insulin stimulates ADH1B enzymatic activity in adipocytes. Following differentiation, cultured adipocytes were starved for 12 h and treated with 1 µM insulin for 1 h (+ Insulin). Untreated cells were used as control (Sham). ADH activity was measured by a colorimetric assay (OD = 450 nm) that yields a proportional color change following ADH catalysis of isopropanol to produce NADH. ADH activity assay was performed in quadruplicate. Data is presented as the mean ± s.e.m. *P < 0.01.

Many factors affect enzyme activity including enzyme concentration and stability, substrate concentration and availability, presence of inhibitors and cellular conditions. To confirm insulin-mediated stimulation of ADH1B protein expression results in increased ADH1B enzymatic activity, we measured alcohol dehydrogenase activity with a colorimetric assay that utilizes isopropanol as a substrate. The results revealed that treatment of lean adipocytes with 1.0 µM insulin for 1 h stimulated a highly significant (P = 0.008) ~ threefold increase in ADH1B activity compared to untreated control, indicating insulin-induced ADH1B expression results in a proportional increase in activity (Fig. 7).

**Figure 8:**
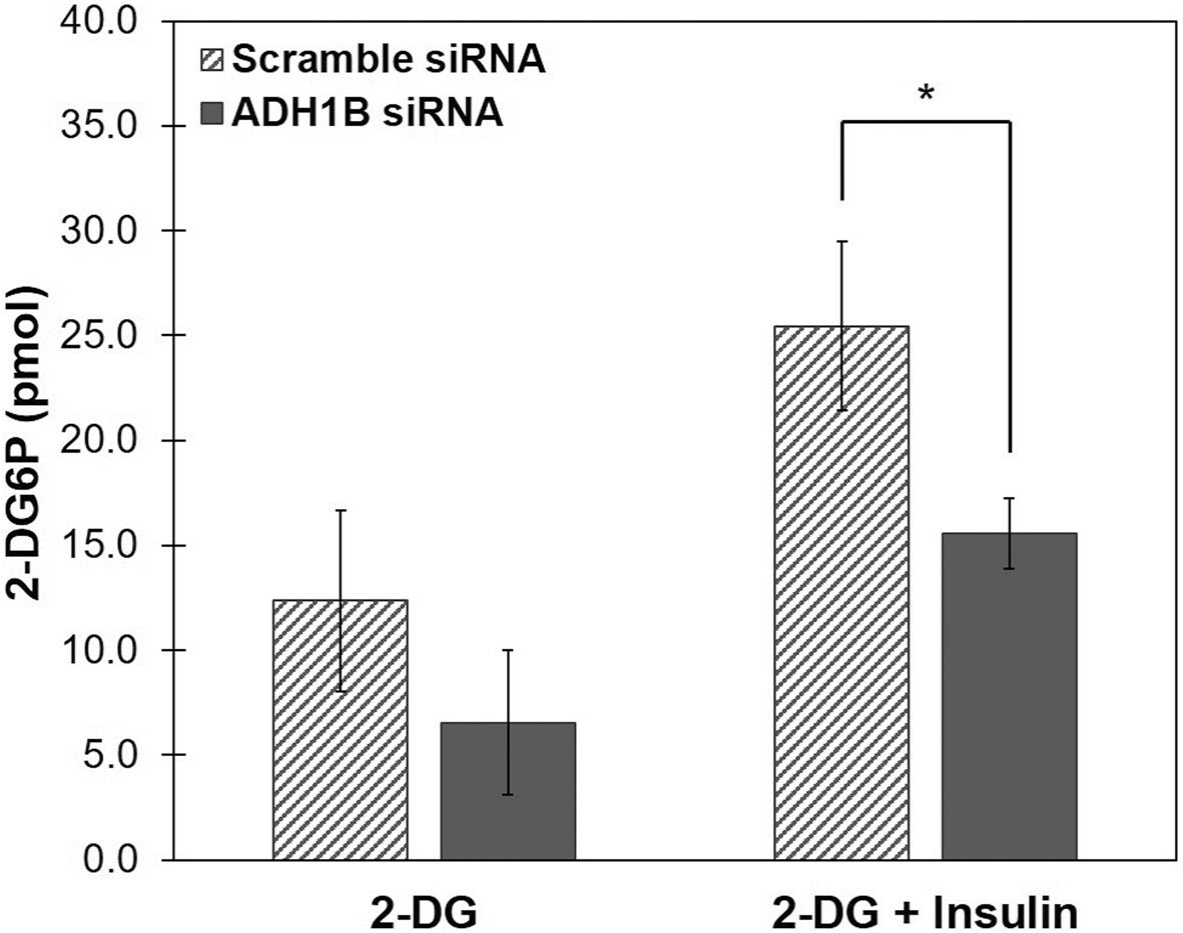
Knockdown of ADH1B yields a decrease in insulin-stimulated glucose uptake within adipocytes. Knockdown of ADH1B was performed by transfection of differentiating pre-adipocytes with ADH1B-specific pooled siRNA or non-targeting (Scramble) control siRNA. Cultured adipocytes were starved for 12 h and then treated with 1 µM insulin (+ Insulin) to stimulate uptake of exogenous 2-deoxyglucose (2-DG) which is metabolized to 2-DG 6 phosphate (2-DG6P). Accumulated 2-DG6P was oxidized to generate a proportional amount of NADPH, yielding an oxidized product that was detected at OD = 412 nm. Data is presented as the mean ± s.e.m. *P < 0.05.

To discover a potential role for insulin-induced ADH1B activity in adipocytes, we used ADH1B-specific pooled siRNA to silence ADH1B protein expression in cultured mature adipocytes. We confirmed the efficacy and specificity of siRNA knockdown in adipocytes by standard Western blot analysis (Supplementary Fig. S12). Pre-adipocytes undergoing differentiation were transfected with non-targeting, i.e. “Scramble”, control siRNA or ADH1B-specific siRNA at Day 9–10 of differentiation. After Day 14, insulin stimulated glucose uptake in mature adipocytes was measured by a cell-based colorimetric assay in which 2-deoxyglucose (2-DG) was taken up and metabolized to 2-DG-6-phosphate. This product was oxidized to produce a proportional amount of NADPH which was quantified. The results showed that deficiency in ADH1B protein expression significantly (P = 0.04) reduced adipocyte glucose uptake following insulin treatment by almost 40% compared to control (Fig. 8). It is possible that ADH1B may have an effect on basal glucose uptake in adipocytes as well. Characterization of the molecular mechanism by which ADH1B may affect glucose uptake in adipocytes requires further study.

## Discussion

We have found that out of 52 human tissues examined, the human *ADH1B* gene overshadowed all others as the most highly expressed in subcutaneous adipose tissue, which is surprising given the well-known critical function of ADH in metabolizing alcohol in the liver. Our previous^22^ and current findings have also revealed that ADH1B gene and protein expression levels are clearly, inversely correlated with BMI (Figs. 1, 4).

A significant (P = 1 × 10^–12^) GWAS signal for association of *ADH1B* coding sequence SNP rs1229984 with BMI was detected in 2019^24^ in Europeans (N = 458,000, UKBiobank) (NHGRI EBI GWAS Catalog, accessed 3/23/2019). In the same study, the same SNP was also associated with cardiovascular disease (N = 8 × 10^–15^) a significant correlate of obesity. A Japanese GWAS (N = 173,430 Japanese males)^25^ in 2017 detected association (P = 1 × 10^–7^) of the same SNP with BMI. Together, all these findings showing the strong association of ADH1B gene and ADH1B expression with BMI and obesity-related traits indicates that ADH1B may be involved in the etiology of obesity, however details of that relationship remain to be uncovered.

The rs1229984 SNP was previously found in multiple studies to be associated with alcohol dependence and upper aerodigestive tract cancer, the latter attributed to the carcinogenic properties of the product of ADH1B oxidation of ethanol (i.e. acetaldehyde)^26–30^. This coding sequence variant, Arg48His, at SNP rs1229984, leads to increased ADH1B enzymatic activity, with more rapid processing of ethanol to toxic acetaldehyde in the liver. It is plausible that the association of SNP rs1229984 with both BMI and alcohol dependence represents independent effects of ADH1B in adipose and liver tissues.

We have begun the investigation into the role of ADH1B in adipocytes in the hopes of determining if ADH1B may contribute to the development of OB/IR. We utilized subcutaneous human pre-adipocyte cell lines that retained their metabolic characteristics, without attenuation, through multiple passages. These cell lines recapitulated central features of gene expression that we previously observed in fat tissue biopsies^19,22^ including expression of key molecules involved in adipogenesis such as ADIPOQ (adiponectin), FABP4, and PPARγ, suggesting they are excellent in vitro models for study. Furthermore, the findings in cultured mature adipocytes indicate that the cellular ADH1B expression phenotype remains unchanged in primary cells derived from tissue and suggest that *ADH1B* expression may be partially regulated by an epigenetic mechanism. This hypothesis is supported by recent findings of Kerr et al*.*^31^ who have shown that *ADH1B* appears to be regulated by an epigenetic mechanism involving CpG-methylation in the 5′ region of the gene. It has also been shown that differences in CpG-methylation between lean and obese are largely maintained between human adipose derived stem cells and mature adipocytes^32^.

Kerr et al.^31^ demonstrated that *ADH1B* was one gene that displayed the highest expression and highest fold increase during differentiation of adipocytes. Our results were similar (Supplementary Fig. S6). Moreover, we revealed ADH1B protein expression also dramatically increased during adipogenesis, whereas ADH1A and ADH1C proteins were not measurably expressed (Fig. 2). This finding suggested ADH1B may be important for adipocyte morphology and function. Whereas Kerr et al^31^ showed that siRNA-knockdown of ADH1B led to decreased differentiation, we found that overexpression of ADH1B during differentiation of pre-adipocytes to cultured mature adipocytes resulted in decreased expression of FABP4. FABP4 is an intracellular lipid transporter highly expressed and secreted by adipocytes which has been shown to regulate PPARγ, a major transcription factor that is critical for adipogenesis and adipose insulin response^33^. Mice deficient in FABP4 do not develop diet-induced insulin resistance or diabetes despite becoming obese, implying FABP4 plays a role in the molecular network that couples obesity with insulin resistance^34^. A study by Wu et al.^35^ also showed that FABP4 can stimulate β-cells to secrete insulin under conditions of obesity to assist in maintaining glucose homeostasis. Given the importance of FABP4 in adipose tissue, ADH1B may also play a significant role in the same cellular pathways as an upstream regulator of FABP4.

We found that ADH1B is involved in the adipocyte response to insulin. The data revealed insulin signaling induces ADH1B protein expression in adipocytes and consequently, it stimulates ADH1B enzyme activity in comparison to untreated control (Figs. 5 and 7). Insulin signaling regulated ADH1B at the transcriptional (Supplementary Fig. S10) and translational level (Supplementary Fig. S11). Moreover, insulin-stimulated ADH1B protein expression occurred via a mechanism requiring activation of AKT kinase, which is involved in many cellular processes including glucose metabolism (Fig. 6).

We next examined the potential effect of ADH1B expression and activity on insulin-stimulated glucose uptake in adipocytes, a key target of insulin action (Fig. 8). Knockdown of ADH1B using ADH1B-specific siRNA led to a 40% reduction in uptake of deoxyglucose (DG) compared with non-targeting scrambled siRNA control. This is the first indication that ADH1B may be involved in insulin action and glucose metabolism in human subcutaneous adipocytes. Further investigation is needed to characterize the exact function of ADH1B in adipose glucose metabolism, and the molecular pathways which regulate its activities.

Previous studies have shown varying effects of gene expression during adipogenesis and, following knockdown, on glucose uptake. The calcineurin-like phosphoesterase domain containing 1 (*CPPED1*) gene expression was unchanged during adipogenesis and decreased in adipose tissue after weight loss^36^. Knockdown of CPPED1 was associated with increased GLUT4 expression and improved insulin-stimulated glucose uptake. The function of *CPPED1* in adipose tissue is unknown. The Receptor Interacting Protein 140 (*RIP140*) gene expression was increased during adipogenesis and decreased in obesity. Its knockdown was associated with increased GLUT4 expression and increased basal glucose transport^37^. The Fat Mass and Obesity Associated gene (*FTO*), which plays an important role in adipocytes, is the single strongest GWAS candidate gene associated with obesity in most worldwide populations, and is the focus of intense research^38^. *FTO* expression increased during adipogenesis^39^. Knockdown of *FTO* expression in the 3T3-L1 mouse adipocyte cell line was associated with a 70% increase in AKT phosphorylation and a 230% decrease in glucose uptake^40^. Thus, the pattern of ADH1B expression during adipogenesis and its association with glucose uptake following knockdown most strongly reflects a pattern similar to that of FTO, implying that FTO and ADH1B may have similar functions in similar cell signaling pathways.

To date there have been no reports of a potential role for ADH1B in human adipocytes or adipose tissue. Our results show, for the first time, that ADH1B is involved in the regulation of FABP4 expression during subcutaneous adipocyte differentiation, an important step in adipose tissue expansion. Our findings also demonstrate, for the first time, that adipocyte ADH1B expression can be regulated by an insulin-mediated mechanism involving AKT phosphorylation. Insulin promoted ADH1B expression and activity, and loss of ADH1B had a negative effect on insulin-stimulated glucose uptake, indicating ADH1B is involved in glucose homeostasis in mature adipocytes. The relationship of ADH1B expression, in adipose tissue and subcutaneous adipocytes, with BMI and insulin activity underscores the importance of its potential role in obesity and insulin resistance. Importantly, our findings point to a potential novel biomarker and molecular pathway underlying obesity and related metabolic disorders which could provide insights that lead to diagnostic and therapeutic personalized interventions.

## Materials and methods

### Reagents

Insulin solution [10 mg/mL], AKT1/2 inhibitor, cycloheximide, actinomycin D, protease inhibitor cocktail, phosphatase inhibitor cocktail I and phosphatase inhibitor cocktail II were purchased from Sigma-Aldrich (St. Louis, MO). Insulin was diluted in basal medium to the necessary concentrations for experimentation as needed. Polybrene [10 mg/mL] was purchased from Millipore (St. Louis, MO) and diluted in medium as needed. Anti-ADH1B, anti-ADH1C, anti-FABP4, and anti-adiponectin antibodies were purchased from Abcam Inc. (Cambridge, MA). Anti-ADH1A was purchased from Origene Technologies, Inc. (Rockville, MD). Anti-DDK, anti-Akt, anti-Phospho-Akt, and anti-PPARγ were purchased from Cell Signaling (Danvers, MA). Anti-GLUT4 was purchased form Bioss, Inc (Boston, MA). Anti-β-actin and IRDye^®^ secondary antibodies were purchased from LI-COR (Lincoln, NE). All reagents were prepared and stored according to the manufacturer’s instructions.

### Pre-adipocyte culture and differentiation

Human subcutaneous pre-adipocytes were purchased from Zen-Bio Inc. Specific lot numbers were selected based on BMI, gender, and age. Information on the donors is available in the Supplementary Information as Supplementary Table S1. Cells were cultured in Pre-adipocyte Medium (Zen-Bio Inc., Research Triangle Park, NC) at 37 °C, 5% CO_2_. For differentiation to mature adipocytes, pre-adipocyte cells were plated at 18,000 cells/cm^2^ and maintained in Pre-adipocyte Medium for 2–4 days until they reached 100% confluency (Day 0). Number of cells was counted using the Vision Cellometer^®^ (Nexcelom Bioscience, Lawrence, MA). Medium was replaced with Adipocyte Differentiation Medium (Zen-Bio Inc.) and cells were incubated for 7 days. Then, cells were fed with Adipocyte Maintenance Medium (Zen-Bio Inc.) every 2–3 days until complete differentiation was achieved at Day 14, according to the manufacturer’s instructions. Cultured mature adipocytes were washed 3 times with sterile DPBS and then starved overnight in serum- and antibiotic-free DMEM/Ham’s F12 medium (1:1) with l-glutamine and 15 mM HEPES (i.e. basal medium) before adding any treatments. For experiments with cycloheximide, differentiated cells were starved and pretreated with 0.178 mM cycloheximide diluted in DMSO 1 h prior to treatment with 0.05 µM insulin for 3 h.

### Oil Red O staining

Differentiated cells were gently washed with sterile DPBS. Then, the cells were fixed in 10% formalin for 60 min. A stock solution of Oil Red O was prepared by mixing 300 mg of Oil Red O powder (Fisher Scientific, Lenexa, KS) with 100 mL of 99% isopropanol. A working solution of 3 parts Oil Red O stock solution and 2 parts deionized water was prepared, filtered through a 0.22 µm syringe filter and allowed to sit for 10 min before use. Next, the fixed cells were gently washed with DPS and incubated with 60% isopropanol for 5 min. After pouring off the isopropanol, the cells were incubated in Oil Red O working solution for 10 min. Lastly, the stained cells were gently rinsed with water until the water ran clear. Unstained and stained cells were visualized using an inverted microscope with an attached digital camera and images of at least 3 random, non-overlapping fields of view were captured. The best representative images were processed using Adobe Photoshop CS5 software. For quantification of lipid accumulation, fixed stained cells were air dried and then the Oil Red O stain was eluted with 100% isopropanol. The OD was measured in triplicate for two biological replicates at 500 nm using a spectrophotometer. Stained empty wells were used as control and 100% isopropanol was used as a blank.

### RNA interference (RNAi)

Cultured adipocytes were transfected with 40 nM of pooled siRNA (SMARTpool ON-TARGETplus, Dharmacon Inc., Lafayette, CO) specific for *ADH1B*, *GAPDH* positive control, or non-targeting (Scramble) negative control according to a protocol modified from Lee et al.^41^. Briefly, during Day 9–10 of differentiation, adipocytes were transfected using Lipofectamine™ 3000 reagent (Invitrogen, Waltham, MA) in basal medium. After ~ 24 h, cells were maintained with Maintenance Medium and starved before treatments as described above.

### Lentiviral transduction

A lentiviral vector for expression of exogenous myc-DDK-tagged human ADH1B and empty vector control were purchased from Origene Technologies, Inc. Lentiviral packaging vectors were received via the nonprofit organization Addgene (Watertown, MA): pMDLg/pRRE (Addgene plasmid # 12251; http://n2t.net/addgene:12251; RRID:Addgene_12251); pRSV-Rev (Addgene plasmid # 12253; http://n2t.net/addgene:12253; RRID:Addgene_12253); and pMD2.G (Addgene plasmid # 12259; http://n2t.net/addgene:12259; RRID:Addgene_12259) were gifts from Didier Trono.

For lentiviral particle production HEK293 cells (Gift from Gilbert Cote) were co-transfected with lentiviral expression vectors and lentiviral envelope and packaging vectors VSV-G, Gag/Pol, and Rev using Lipofectamine™ 3000 reagent in Opti MEM I reduced-serum medium (Invitrogen) using manufacturer’s instructions. After 6 h incubation, the medium was changed. After 24 h incubation, the medium containing viral particles was collected, stored at 4 °C, and replaced with fresh medium. After a second 24 h incubation, the medium was collected. The two fractions were pooled and filter sterilized using a 0.45 µm syringe filter. Then, the viruses were concentrated by centrifugal ultrafiltration using an Amicon^®^ Ultra-15 centrifugal filter (Millipore) at 1000×*g*, 4 °C.

For transduction, pre-adipocytes were plated at 18,000 cells/cm^2^ and maintained in Pre-adipocyte Medium until confluency. Next, cells were incubated in 250 µL lentivirus, 250 µL fresh medium, and 8 µg/mL polybrene for 12 h. Then, medium was aspirated, cells were washed with PBS, and incubated in fresh medium. The following day differentiation was initiated as described above. On Day 10 of differentiation, cells were transduced with lentivirus a second time and differentiation continued as described above.

### Quantitative polymerase chain reaction (qPCR)

Total RNA was isolated using the RNeasy Plus Mini kit (Qiagen, Hilden, Germany). RNA concentrations were measured on a NanoDropTM spectrophotometer and qPCR was performed using TaqMan^®^ Gene Expression Assays for human *ADH1A*, *ADH1B*, *ADH1C*, and *GAPDH* endogenous control (ThermoFisher Scientific, Waltham, MA). qPCR analysis was performed in triplicate or quadruplicate. Data is presented as mean ± standard deviation (s.d.). For experiments with actinomycin D, cells were differentiated as described above. Then, adipocytes were pretreated with 0.3 µg/mL actinomycin D diluted in medium for 0.25 h before addition of 0.05 µM insulin. Cells were incubated for up to 4 h in actinomycin D ± insulin. Total RNA was collected and quantified as described and qPCR analysis was performed in quadruplicate for two biological replicates. Data is presented as mean ± s.e.m. Results were analyzed with Microsoft Excel software by applying the equal variance t Test. P ≤ 0.05 was considered statistically significant.

### Western blot analysis

Cell lysates were prepared with Pierce^®^ RIPA Buffer (ThermoFisher Scientific) containing protease and phosphatase inhibitors and the amount of total protein in the cell lysates was quantified using the Pierce^®^ BCA assay (Thermofisher Scientific). Equal amounts (5–20 µg) of total protein lysates were resolved using SDS-PAGE. The samples were then transferred onto a PVDF or nitrocellulose membrane using an electroblotting method. For two-color fluorescent immunoblotting, the membrane was incubated overnight at 4 °C with primary antibodies from two different host species for the target protein and the loading control, followed by incubation in the dark with infrared fluorescent dye (IRDye)-conjugated secondary antibodies. One secondary antibody was labeled with a 700 nm channel dye and the other with an 800 nm channel dye. Fluorescence intensity was measured using the LI-COR Odyssey CLx infrared imaging system (Resolution: 169 µm; Intensity: auto; Quality: lowest). LI-COR iS Image Studio software was used for image modification (channel realignment, brightness, contrast, color inversion) and data analysis (band intensity and normalization). Relative expression for a test sample was determined as: Normalized Target Signal/Normalized β-actin Signal. Western blots were performed at least three times. Samples derived from the same experiment were processed in parallel for immunoblotting with multiple antibodies or immunoblots were stripped with RestoreTM fluorescent western blot stripping buffer (Thermofisher Scientific) as needed. Data were presented as mean expression ± s.e.m. Results were analyzed with Microsoft Excel software by applying the equal variance t Test. P ≤ 0.05 was considered statistically significant. Cropped Western blot images were processed using Adobe Photoshop CS5 software.

### Alcohol dehydrogenase activity assay

Cell lysates were prepared with RIPA buffer containing protease and phosphatase inhibitors. Activity of ADH was quantified according to the manufacturer’s instructions (BioVision Inc., Milpitas, CA). Briefly, equal amounts of total protein were diluted in ice-cold Assay Buffer up to 50 µl/well of a 96-well plate. Reaction mix, containing isopropanol substrate, was added to wells containing experimental samples, positive controls, and NADH standards. The plate was incubated for 3 min at 37 °C and the OD was measured at 450 nm (A_0_) using a microplate reader. The plate was re-incubated for an additional 30 min to 2 h at 37 °C and OD was measured again at 450 nm (A_1_) by a kinetic method every 5 min. The NADH standard curve was read in Endpoint Mode at the end of the incubation time, and the 0 standard value was subtracted from all values. ADH activity was calculated as: (B/(ΔT × V)) × Sample Dilution = mU/mL, where B was the amount of NADH (nmol) generated by ADH during ΔT (ΔT = T_2_ – T_1_). T was the reaction time (min), and V was the volume (mL) of sample used. B was determined by applying the ΔOD (ΔOD = A_1_ – A_0_) to the NADH standard curve. The experiment was performed four times, and data were presented as the mean ± s.e.m. Results were analyzed with Microsoft Excel software by applying the equal variance t Test. P ≤ 0.05 was considered statistically significant.

### Glucose uptake assay

The glucose uptake assay (Abcam, Inc.,Cambridge, MA) was performed according to the manufacturer’s instructions. Briefly, pre-adipocytes were differentiated to adipocytes as described and maintained for an additional 4 days. Cultured cells were then starved overnight as described. Cultured cells were washed and starved in KRPH + 2% BSA buffer. Then 1 µM insulin and 2-deoxyglucose (2-DG) were added to activate glucose transporters to take up 2-DG and metabolize it to 2-DG-6-phosphate (2-DG6P). The accumulated 2-DG6P was oxidized to NADPH which generated an oxidized substrate that was measured at OD_412nm_ using a microplate reader. A 2-DG6P standard curve was also measured at the same time. Absorbance values were normalized to the mean “blank” absorbance value. Cells not treated with insulin or 2-DG were used as sample background controls. 2-DG uptake [µM] was calculated as: (Calculated concentration of 2-DG6P/Sample volume) × Sample dilution. The experiment was performed three times, and data were presented as the mean ± s.e.m. Results were analyzed with Microsoft Excel software by applying the equal variance t Test. P ≤ 0.05 was considered statistically significant.

### Aggregation of values with meta-analysis tool

We requested statistical values from the following sources of samples and method for detection of RNA species: (1) TwinsUK Repository (RNAseq) [Small, K., Glastonbury C., et al.]^42^, N = 766 European females (40% monozygotic and 60% dizygotic pairs), BMI range 16–47, median 25), Age 38–84; 9 exons tested separately. (2) NIDDK Phoenix, (GeneChip Human Exon Arrays) [Hanson, R.L., Hsueh W–C., et al.] N = 200 Pima Indians, Nominal P values corrected for age and sex (unpublished). (3) Wake Forest University, (Beadarrays), [Das, S.K., Langefeld, C.D., et al.], SI = insulin sensitivity index, measured by the frequently sampled iv glucose tolerance test (FSIGT), Sharma et al.^43^ We performed an aggregation of standardized beta values from the correlation (using meta-analysis statistical tools) between *ADH1B* expression with BMI and blood glucose. We collected BMI and glucose data from, respectively, the TwinsUK study, (n = 448 and 353), African Americans (n = 251, BMI only), Pima Indians (n = 210 and 77) and Mexican Americans (n = 54 for both traits). A random effects model was used due to the heterogeneity of the samples (I^2^ = 65.2% for BMI and 86.1% for glucose). These analyses were performed with “metan” command from STATA 15.1 (StataCorp LLC, TX).

## Supplementary Information

Supplementary Information.

## Data Availability

Data generated and analyzed during this study are presented in this published article (and its Supplementary Information files). All datasets are available from the corresponding authors on reasonable request.
