# Supplementary material for: Further evidence supporting a potential role for ADH1B in obesity
Source: Sci Rep. 2021 Jan 21;11:1932. doi: 10.1038/s41598-020-80563-z (PMC7820614; doi:10.1038/s41598-020-80563-z)
Supplement: Supplementary file 1 — Supplementary Information. [file 41598_2020_80563_MOESM1_ESM.pdf]

Further evidence supporting a potential role for ADH1B in obesity

Liza D. Morales, Douglas T. Cromack, Devjit Tripathy, Marcel Fourcaudot, Satish Kumar, Joanne E. Curran, Melanie Carless, Harald H. H. Göring, Shirley L. Hu, Juan Carlos Lopez-Alvarenga, Kristina M. Garske, Päivi Pajukanta, Kerrin S. Small, Craig A. Glastonbury, Swapan K. Das, Carl Langefeld, Robert L Hanson, Wen-Chi Hsueh, Luke Norton, Rector Arya, Srinivas Mummidi, John Blangero, Ralph A. DeFronzo, Ravindranath Duggirala, Christopher P. Jenkinson

BMI	GENDER	AGE	DIABETIC
23.3	Female	40	No
21.6	Female	38	No
26.2	Female	48	No
38.0	Female	39	No
53.5	Female	34	Yes

Supplementary Table S1. Pre-adipocyte cell line donor information. Lot composition (1 donor per lot) provided by manufacturer on Certificate of Analysis for cryopreserved vial of human subcutaneous pre-adipocytes.

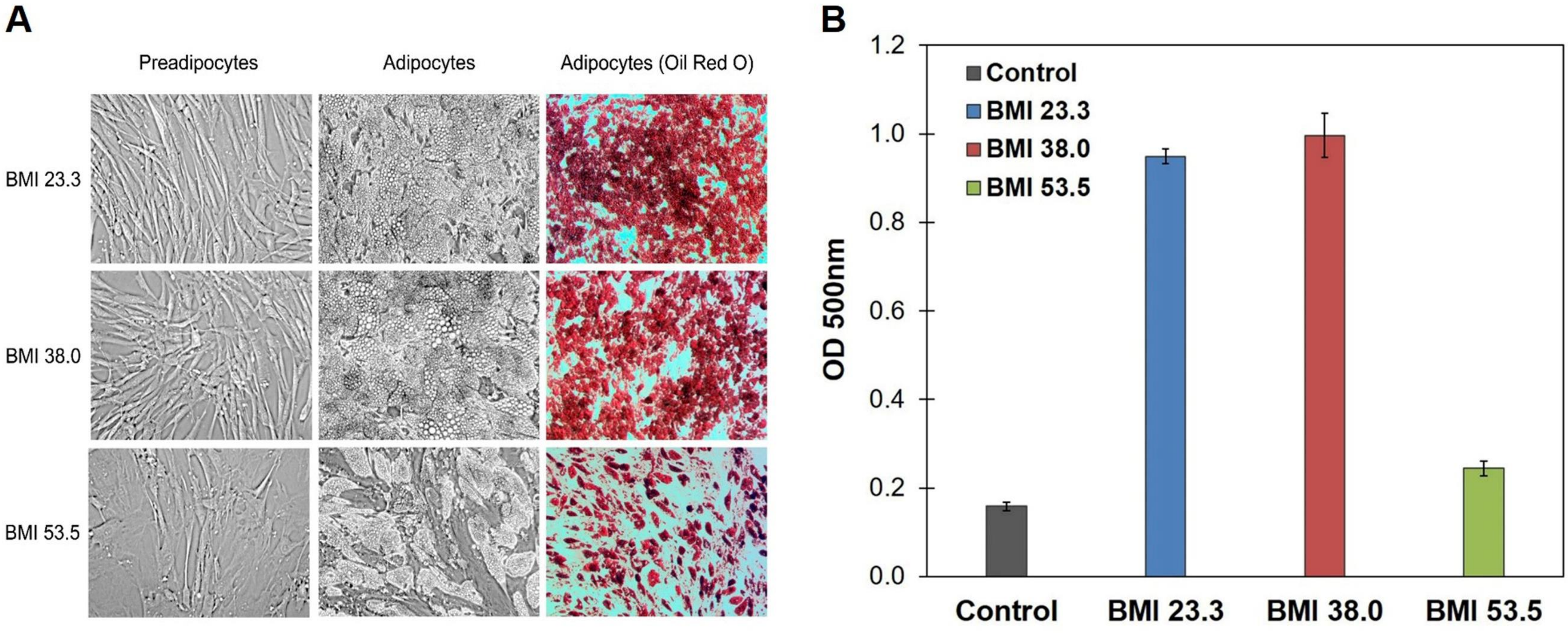

Supplementary Figure S1. Differentiation of human subcutaneous preadipocytes. Pre-adipocytes derived from lean (BMI < 30 kg.m^{-2}) or obese (BMI ≥ 30 kg.m^{-2}) donors were cultured in Pre-adipocyte Growth Medium. To initiate differentiation, conditioned growth medium was replaced with Adipocyte Differentiation Medium and then cells were cultured for 7 days. On day 7, the conditioned medium was replenished with Adipocyte Maintenance Medium; medium was replenished every 2 – 3 days for an additional 7 days. (A) Representative images of human subcutaneous preadipocytes and cultured mature adipocytes in culture (100x magnification) or fixed and stained with Oil Red O (40x magnification). (B) Quantification of lipid accumulation. Oil Red O stain was eluted from fixed, stained cells and the intensity of the color was measured at OD = 500 nm. Data is presented as the mean ± s.e.m.

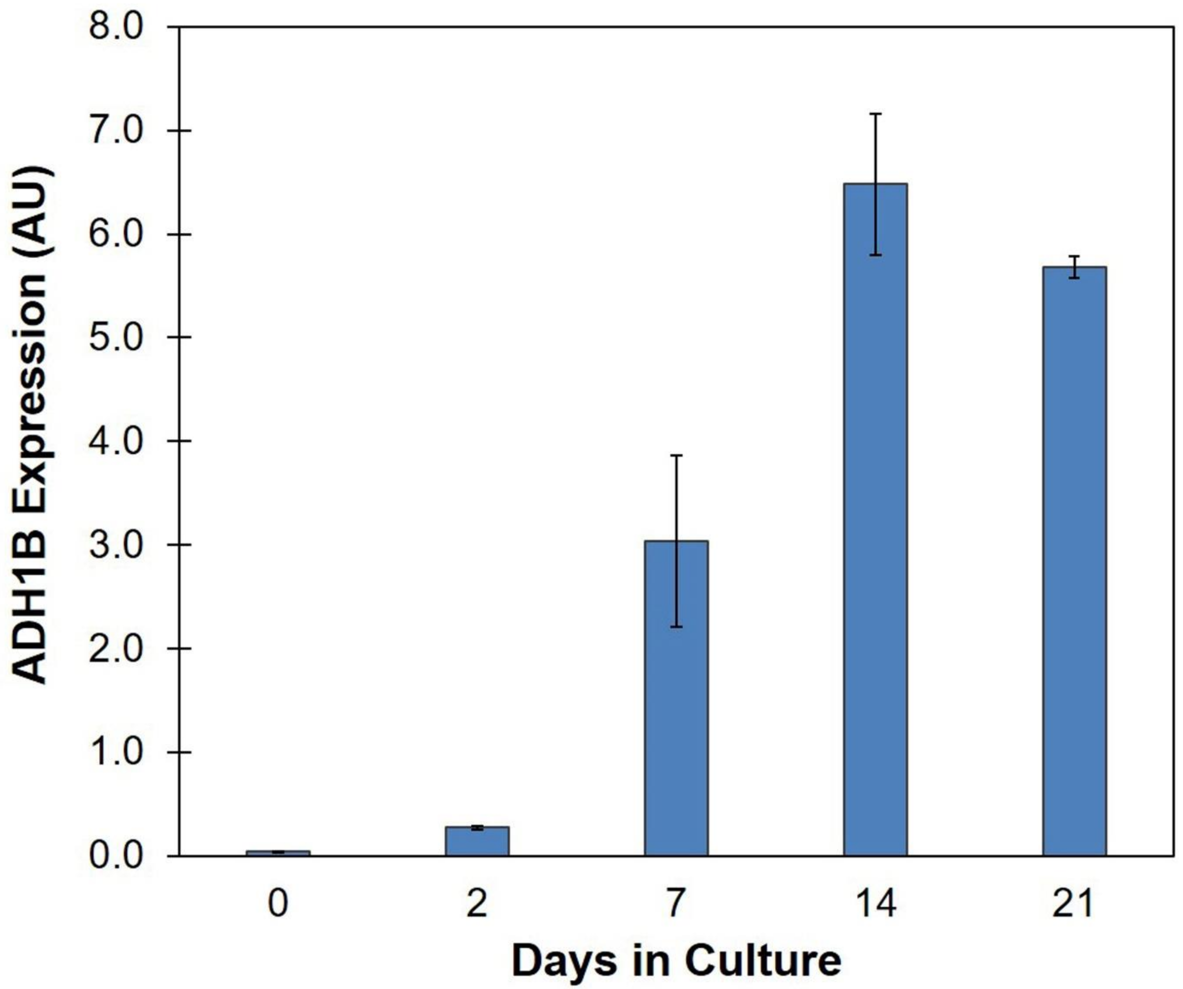

Supplementary Figure S2. Cultured mature adipocytes express a basal level of ADH1B. Total protein was isolated from human subcutaneous preadipocytes (Day 0) and mature adipocytes (Day 14 - 21) at the indicated time points, resolved by SDS-PAGE, and immunoblotted with an antibodies specific for ADH1B and β-Actin loading control. Fluorescence intensity was quantified using the LI-COR Odyssey CLx infrared imaging system (Resolution: 169µm; Intensity: auto; Quality: lowest) and normalized to β-Actin control. Western blot analysis was performed in triplicate. Data is presented as the mean ± s.e.m. AU, Arbitrary Units

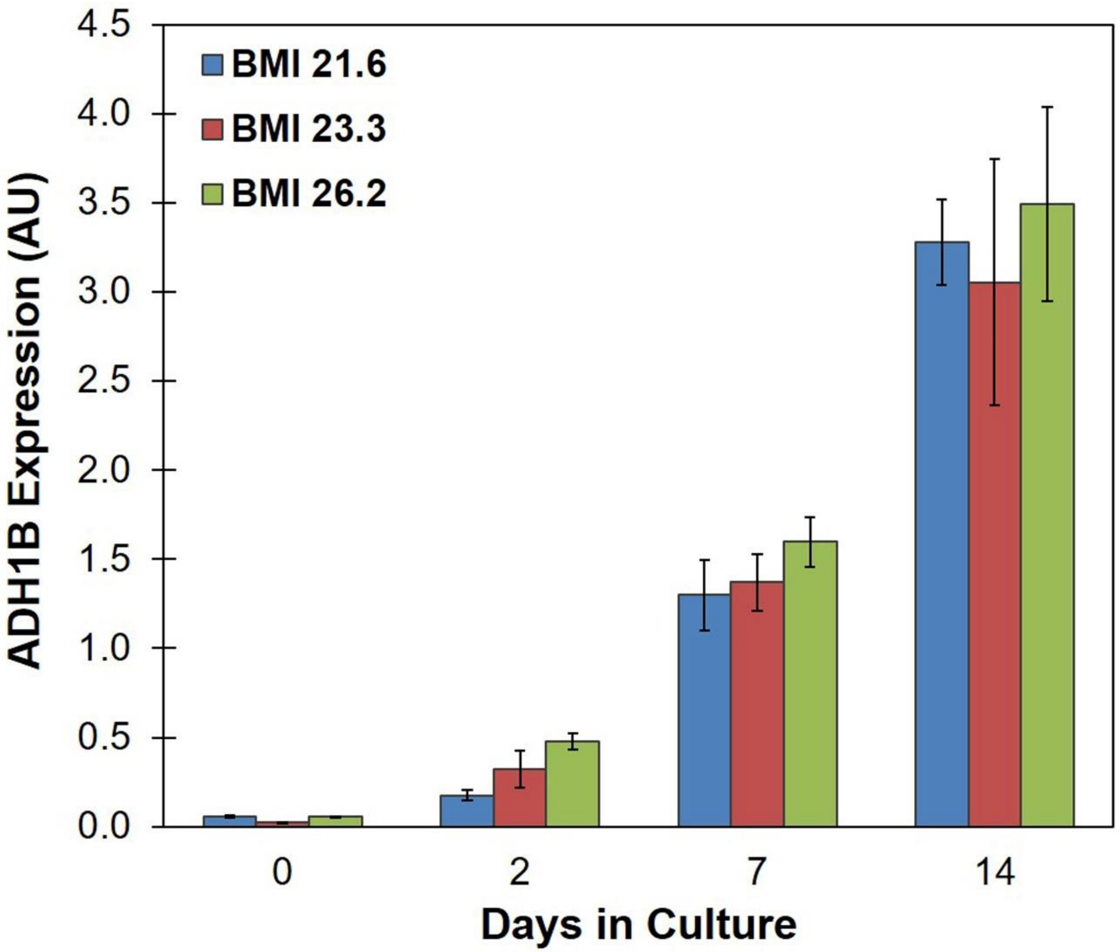

Supplementary Figure S3. Expression levels of ADH1B in differentiating pre-adipocytes derived from different lean donors. Total protein was isolated from human subcutaneous pre-adipocyte cell lines derived from three different donors with similar age, gender, and BMI during differentiation to mature adipocytes. Donor information can be found in Supplementary Table S1. Total protein was isolated at the indicated time points during differentiation, resolved by SDS-PAGE, and immunoblotted with antibodies specific for ADH1B and β-Actin loading control. Fluorescence intensity was quantified using the LI-COR Odyssey CLx infrared imaging system (Resolution: 169μm; Intensity: auto; Quality: lowest) and normalized to β-Actin control. Western blot analysis was performed in triplicate. Data is presented as the mean ± s.e.m. AU, Arbitrary Units

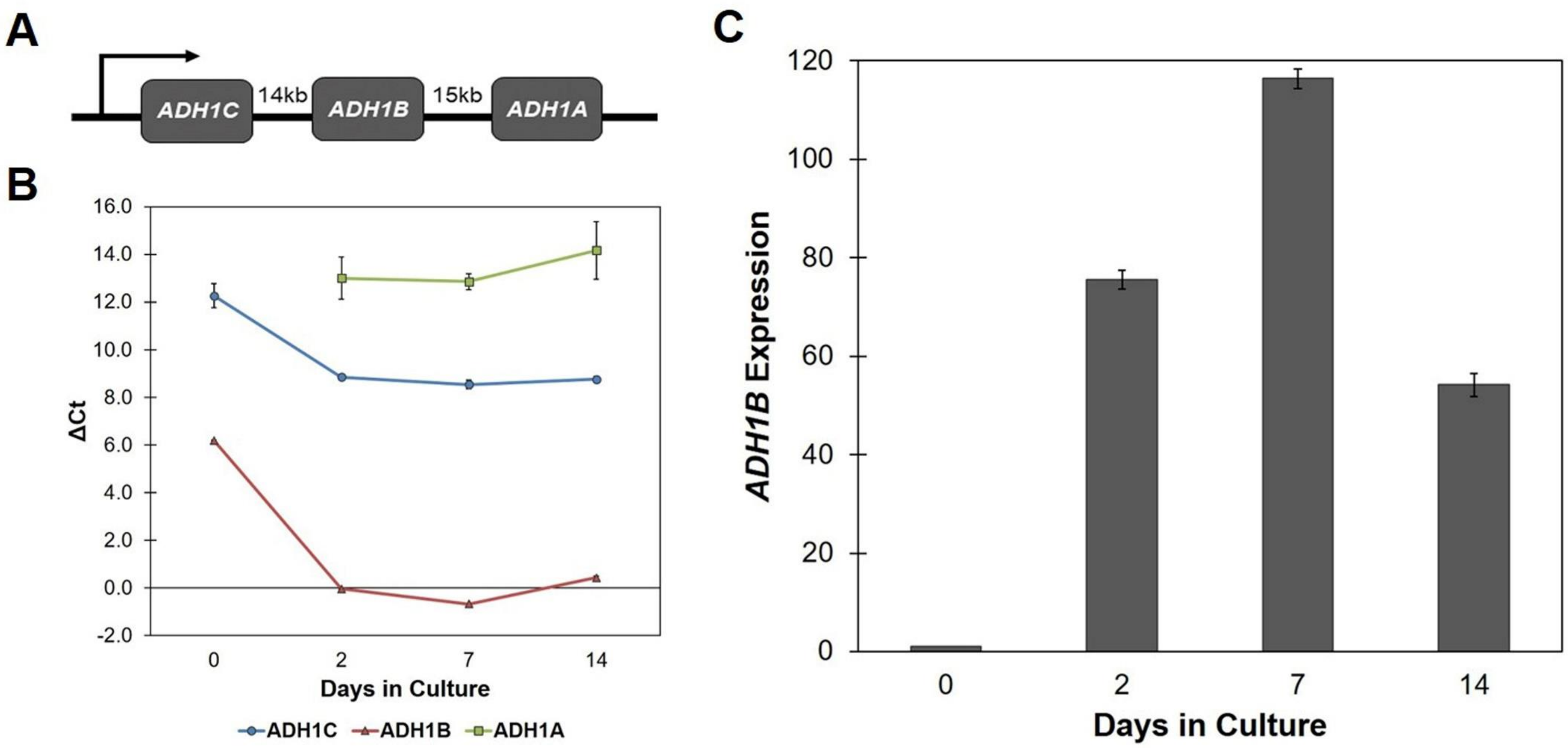

Supplementary Figure S4. Expression levels of *ADH1* mRNA in differentiating pre-adipocytes. (A) Positions of the three human *ADH1* gene isoforms on chromosome 4q. The genes are shown in the direction in which they are transcribed which is opposite to their orientation on the chromosome. The distances between the genes are shown. (B) Quantification of *ADH1C*, *ADH1B*, and *ADH1A* mRNA expression levels in pre-adipocytes and adipocytes during differentiation. Total RNA was isolated at the indicated time points during differentiation. qPCR analysis was performed in triplicate. GAPDH was utilized as endogenous control. Data is presented as the mean ± s.d. AU, Arbitrary Units. Low ΔCt represents high mRNA expression. (C) Expression of *ADH1B* after initiation of differentiation compared to pre-adipocytes (Day 0). Fold increase was calculated as $2^{(-\Delta\Delta Ct)}$. Data is presented as the mean ± s.d.

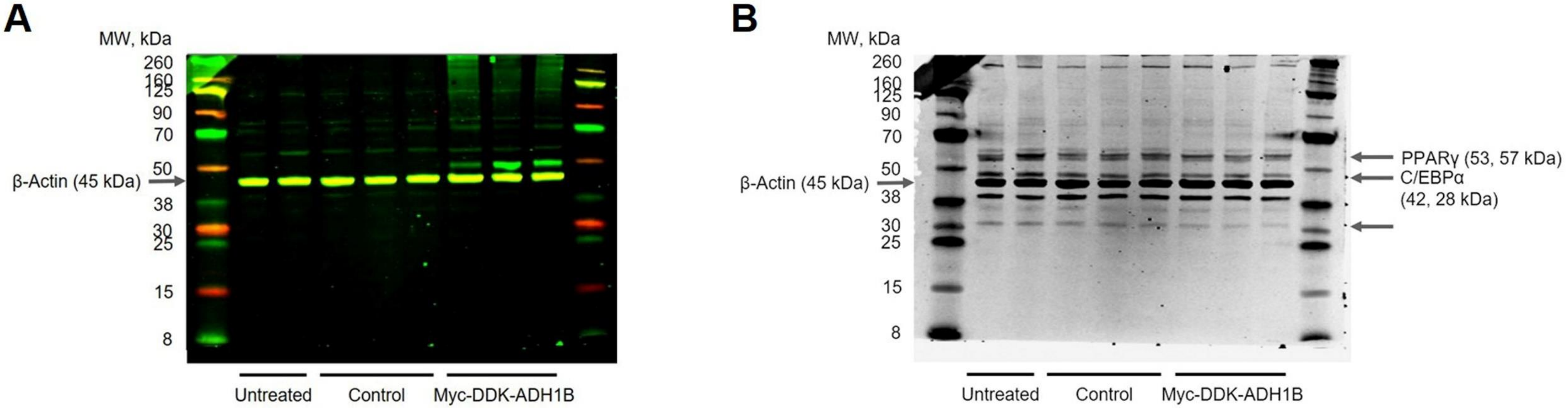

Supplementary Figure S5. Overexpression of ADH1B in cultured human subcutaneous adipocytes. Two-color fluorescent immunoblot analysis of cell lysates from biological replicates of lean (BMI < 30 kg.m^{-2}) adipocytes following differentiation and transduction with lentiviruses. Pre-adipocytes were transduced with lentivirus containing empty vector (Control) or vector encoding myc-DDK-tagged human ADH1B on Day 0 and Day 10 of differentiation in triplicate. Untreated pre-adipocytes were differentiated in duplicate and utilized as an additional control. Total protein was collected following complete differentiation, resolved by SDS-PAGE, and immunoblotted with antibodies specific for (A) DDK tag, (B) PPARγ or C/EBPα. β-Actin was utilized as loading control. Fluorescence intensity was measured using the LI-COR Odyssey CLx infrared imaging system (Resolution: 169µm; Intensity: auto; Quality: lowest).

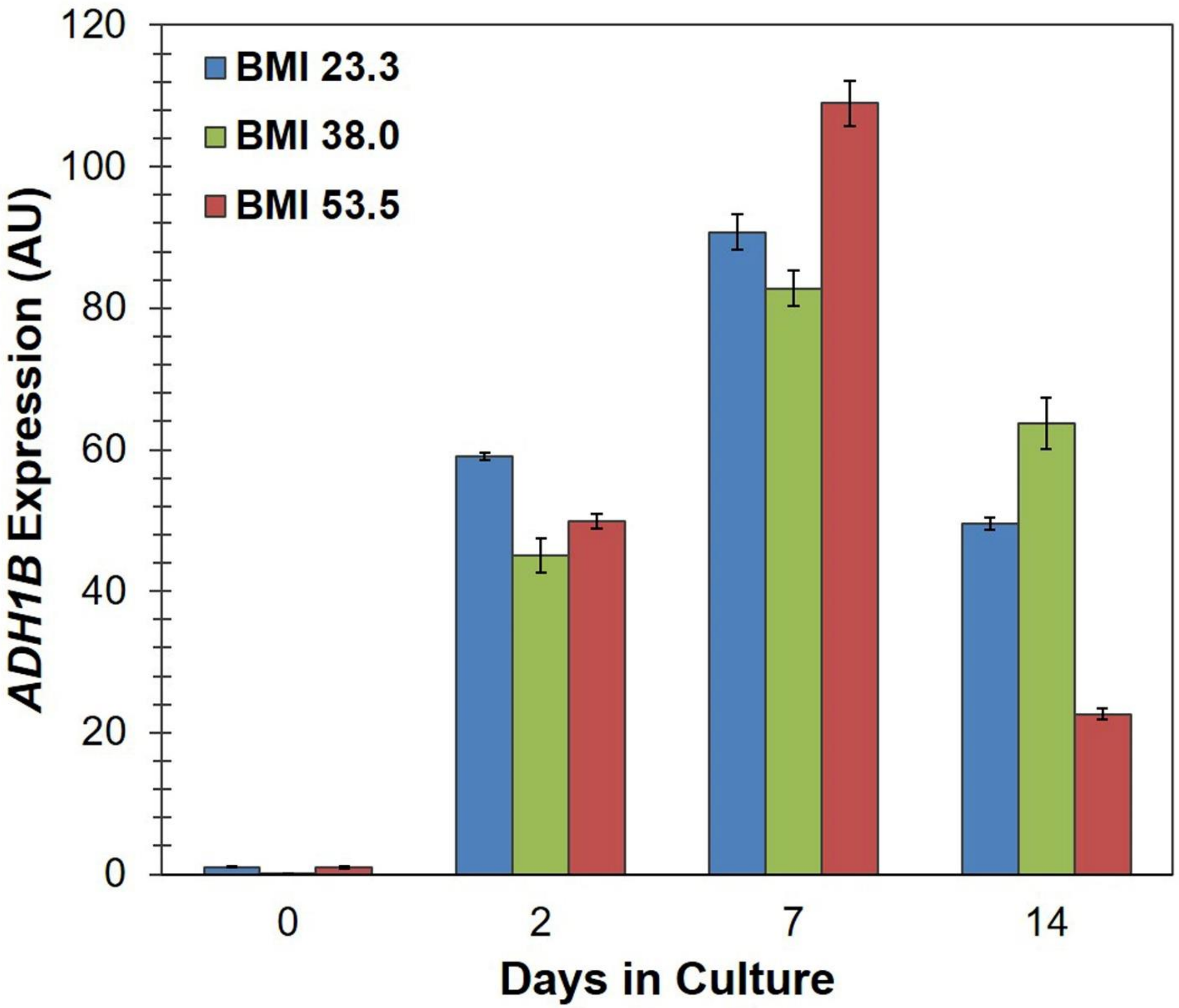

Supplementary Figure S6. Expression levels of *ADH1B* mRNA during adipogenesis. Quantification of *ADH1B* mRNA expression levels in lean (BMI < 30 kg.m^{-2}) or obese (BMI ≦ 30 kg.m^{-2}) pre-adipocytes undergoing differentiation compared to pre-adipocytes (BMI 23.3 kg.m^{-2}, Day 0). Total RNA was isolated at the indicated time points during adipogenesis. qPCR analysis was performed in quadruplicate. GAPDH was utilized as endogenous control. Data is presented as the mean ± s.d. AU, Arbitrary Units

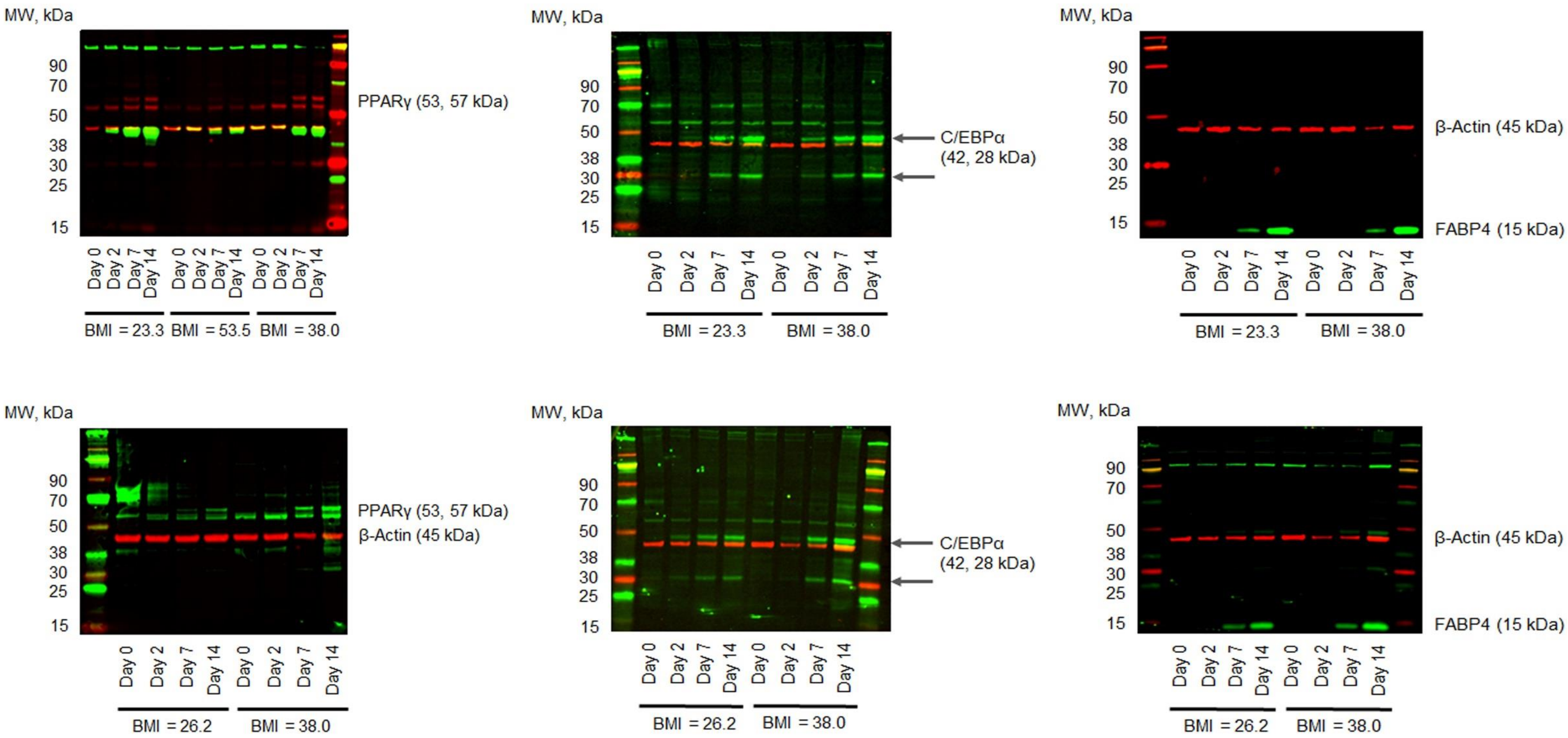

Supplementary Figure S7. Pre-adipocytes from lean and healthy obese donors undergo comparable levels of adipogenesis. Representative images of two-color fluorescent immunoblot analysis of cell lysates from lean (BMI < 30 kg.m^{-2}) or obese (BMI = 38 kg.m^{-2}) adipocytes during adipogenesis. Total protein was collected at the indicated time points during differentiation, resolved by SDS-PAGE, and immunoblotted with antibodies specific for the major differentiation markers PPARγ, C/EBPα, FABP4 and β-Actin loading control. Fluorescence intensity was measured using the LI-COR Odyssey CLx infrared imaging system (Resolution: 169µm; Intensity: auto; Quality: lowest).

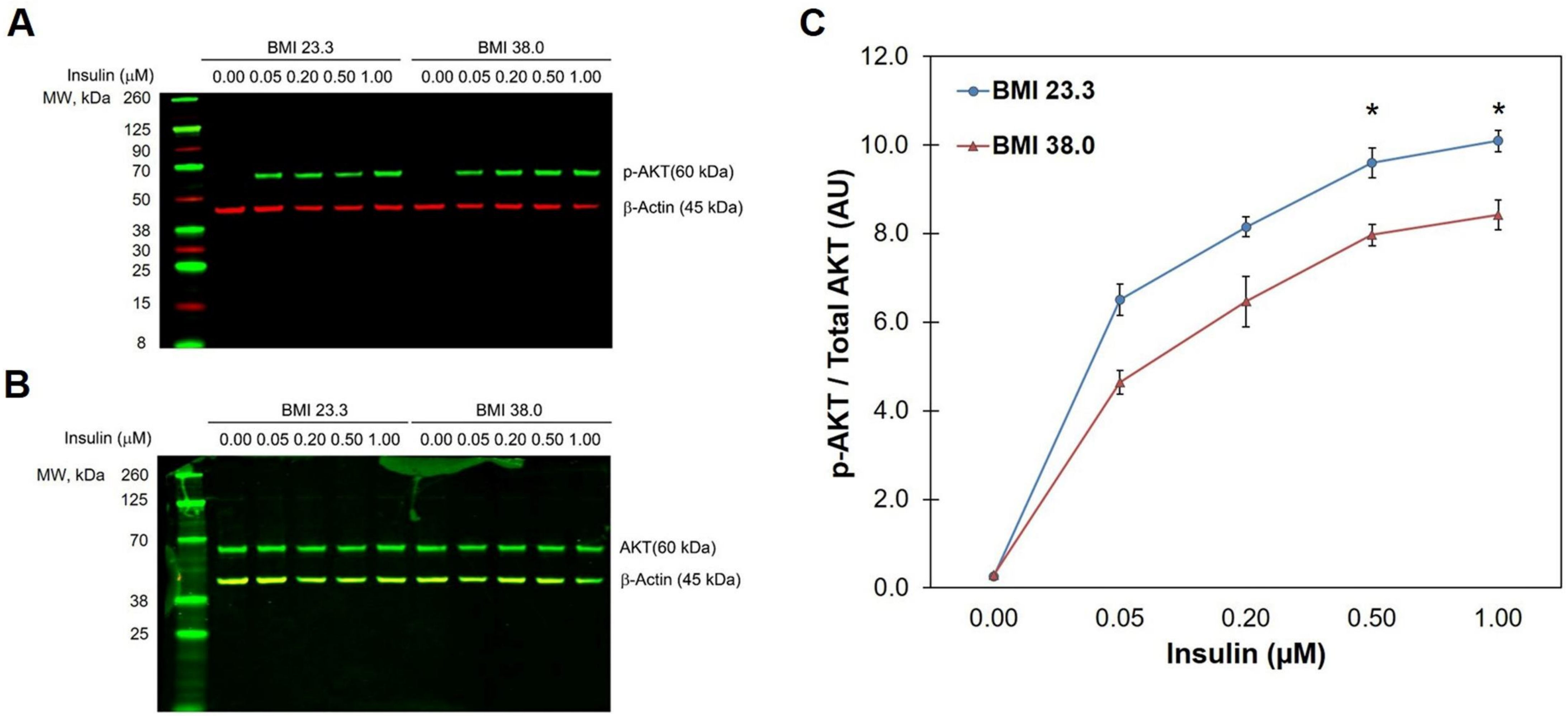

Supplementary Figure S8. Adipocytes derived from obese donor adipose tissue display insulin resistance *in vitro*. **(A,B)** Representative images of two-color fluorescent immunoblot analysis of cell lysates from lean (BMI 23.3 $kg.m^{-2}$) or obese (BMI 38.0 $kg.m^{-2}$) adipocytes following treatment with insulin. Cells were starved for 12 h and then treated with the indicated dose of insulin for 1 h. Total protein was isolated, resolved by SDS-PAGE, and immunoblotted with antibodies specific for **(A)** phosphorylated AKT (p-AKT) and **(B)** total AKT. β-Actin was used as loading control. **(C)** Quantitative analysis of AKT activation (Ratio of p-AKT to AKT expression). Samples derived from the same experiment were processed in parallel. Western blot analysis was performed in triplicate. Fluorescence intensity was measured using the LI-COR Odyssey CLx infrared imaging system (Resolution: 169µm; Intensity: auto; Quality: lowest). Data is presented as the mean ± s.e.m. *P ≤ 0.01 AU, Arbitrary Units

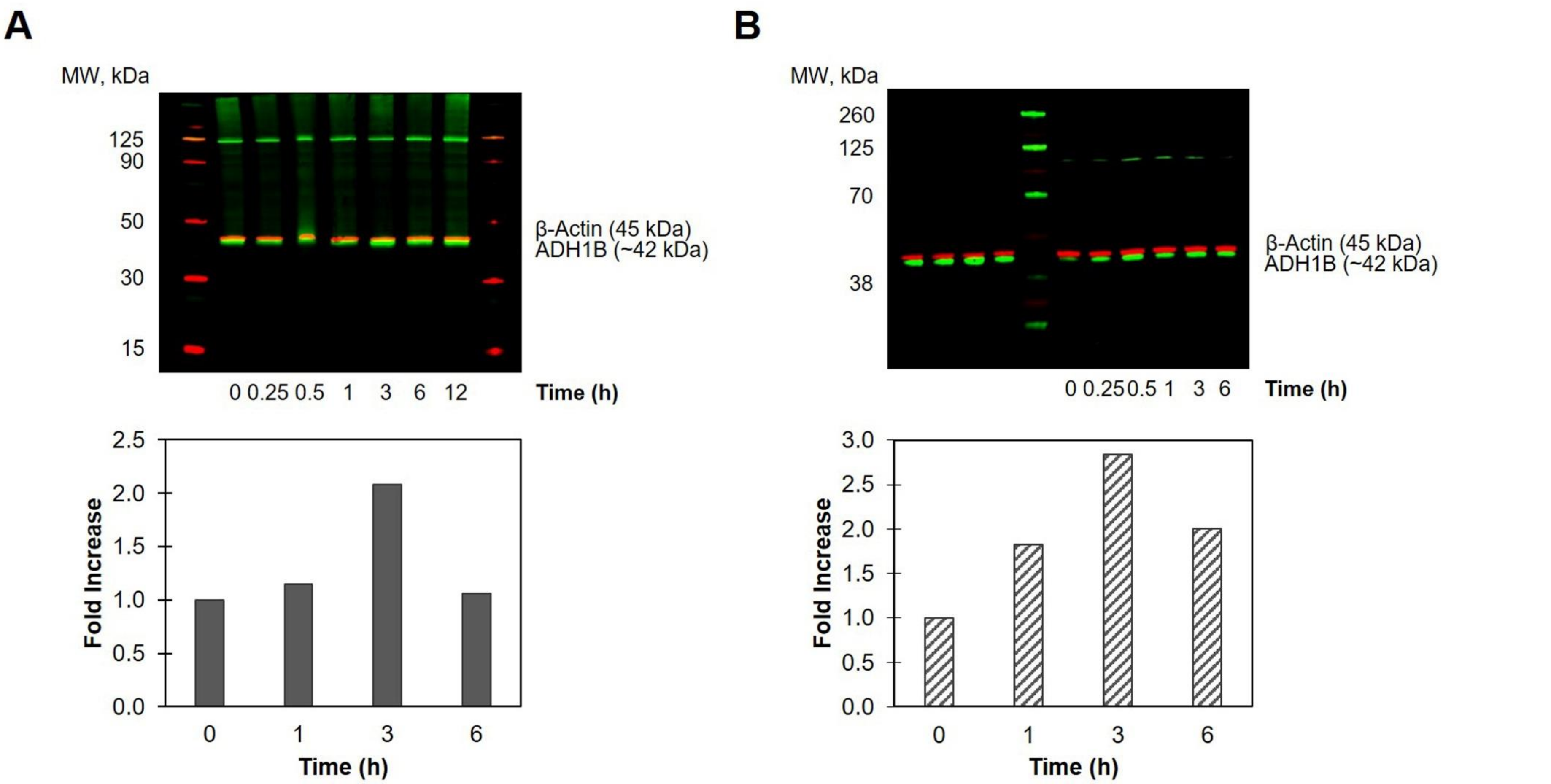

Supplementary Figure S9. Effect of treatment time on insulin-induced ADH1B expression. (A,B) Representative images of two-color fluorescent immunoblot analysis of cell lysates from lean (BMI < 30 kg.m^{-2}) cultured adipocytes following incubation with insulin for increasing periods of time. Cells were starved for 12 h and then treated with (A) 0.05 µM or (B) 1.0 µM insulin for the indicated time points. Total protein was isolated, resolved by SDS-PAGE, and immunoblotted with antibodies specific for ADH1B (800 nm channel) and β-Actin (700 nm channel) loading control. Fluorescence intensity was measured using the LI-COR Odyssey CLx infrared imaging system (Resolution: 169µm; Intensity: auto; Quality: lowest). Fold increase was calculated as ADH1B expression at T_n divided by ADH1B expression at T_0, where T = time

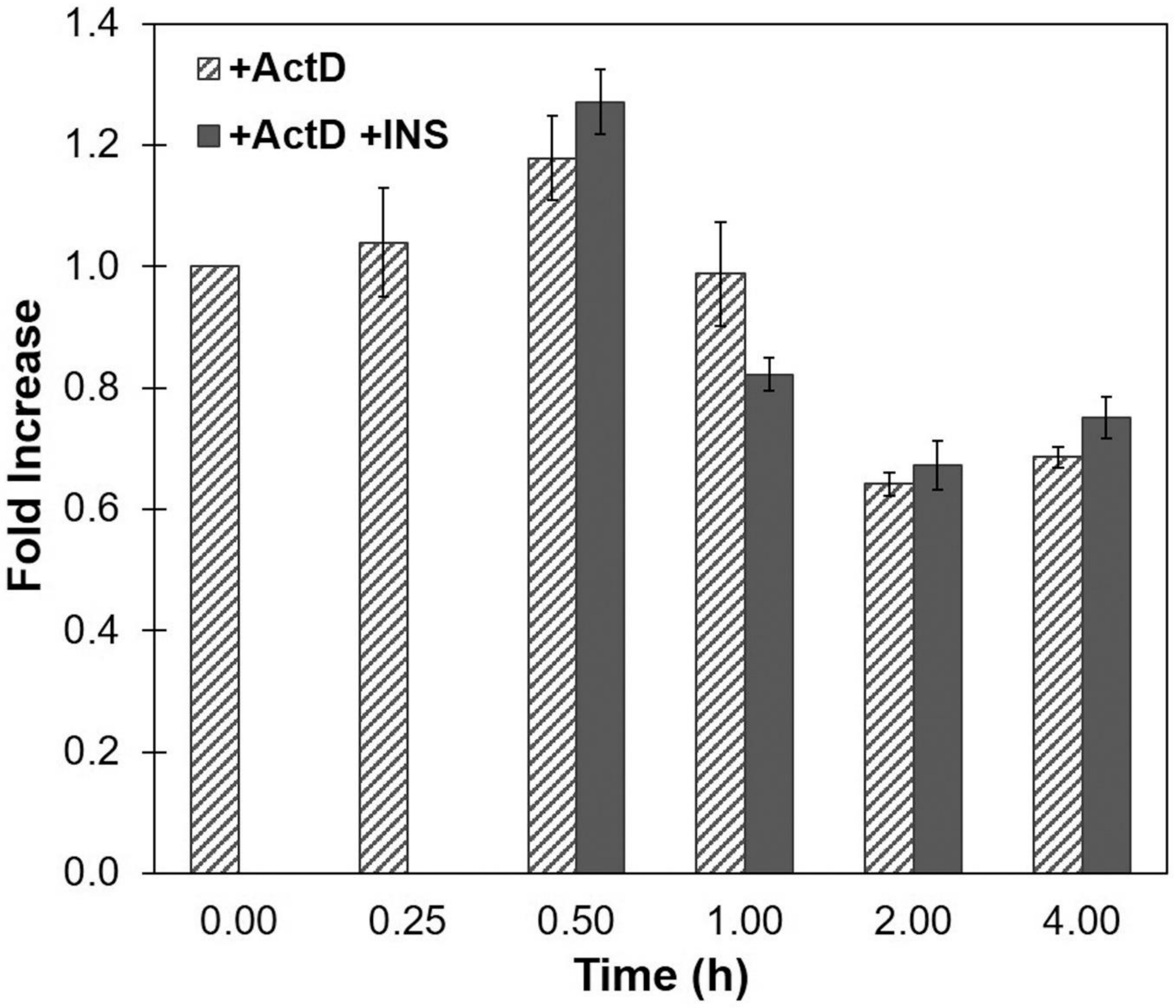

Supplementary Figure S10. Insulin signaling regulates *ADH1B* mRNA expression at the transcriptional level. Quantification *ADH1B* mRNA expression levels in the presence or absence of insulin treatment following RNA synthesis inhibition. Cultured mature adipocytes were pretreated with 0.3 µg/mL actinomycin D (+ActD) before addition of 0.05 µM insulin (+INS). Untreated cells (0 h) were utilized as control. Total RNA of two biological replicates was isolated at the indicated time points following ActD treatment and used in qPCR analysis run in quadruplicate. GAPDH was utilized as endogenous control. Data is presented as the mean fold increase ± s.e.m.

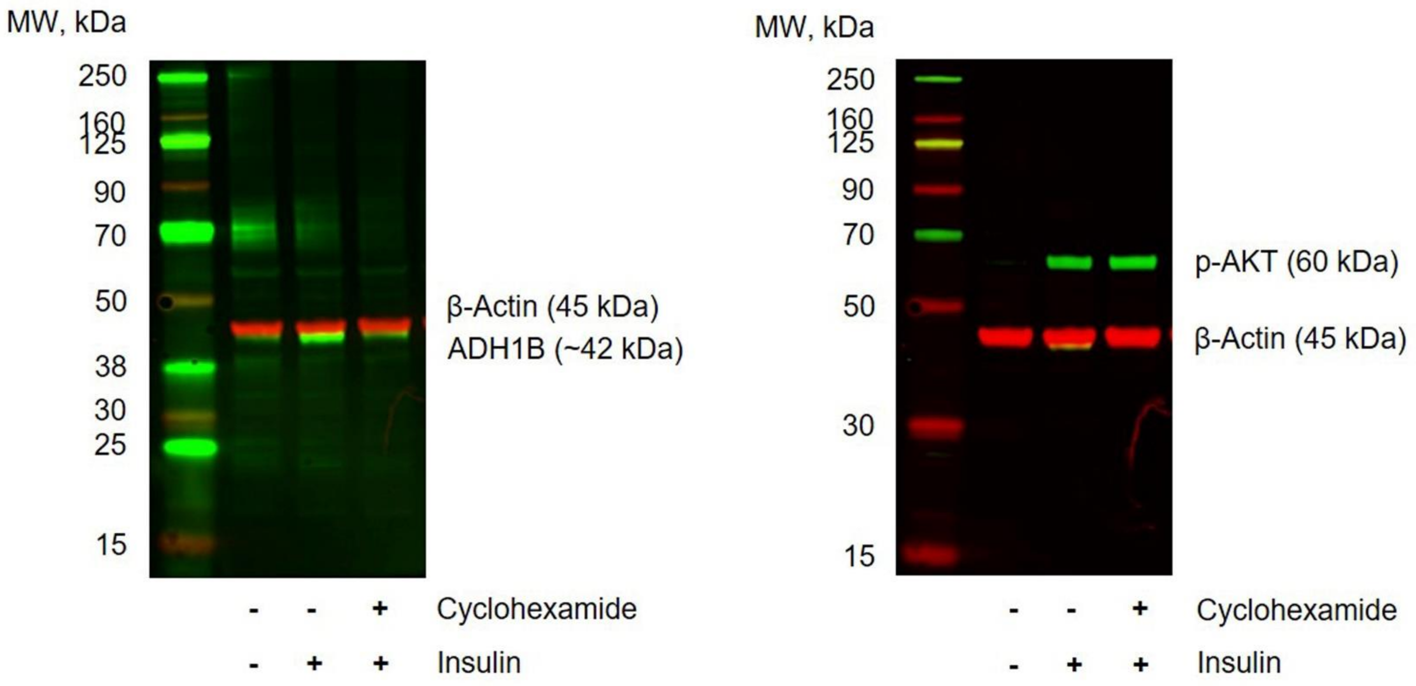

Supplementary Figure S11. Insulin signaling regulates ADH1B expression at the translational level. Two-colored fluorescent immunoblot analysis of cell lysates from lean (BMI < 30 kg.m^{-2}) adipocytes with and without treatment with cyclohexamide and/or insulin. Cultured adipocytes were starved for 12 h and then treated with 0.178 mM cyclohexamide (+) for 1 h prior to treatment with 0.05 µM insulin (+) for 3 h. Untreated cells (- Insulin, - Cyclohexamide) were utilized as controls. Total protein was isolated, resolved by SDS-PAGE, and immunoblotted with antibodies specific for ADH1B (800 nm channel), phosphorylated AKT (p-AKT, 800 nm channel) and β-Actin (700 nm channel) loading control. Fluorescence intensity was measured using the LI-COR Odyssey CLx infrared imaging system (Resolution: 169µm; Intensity: auto; Quality: lowest).

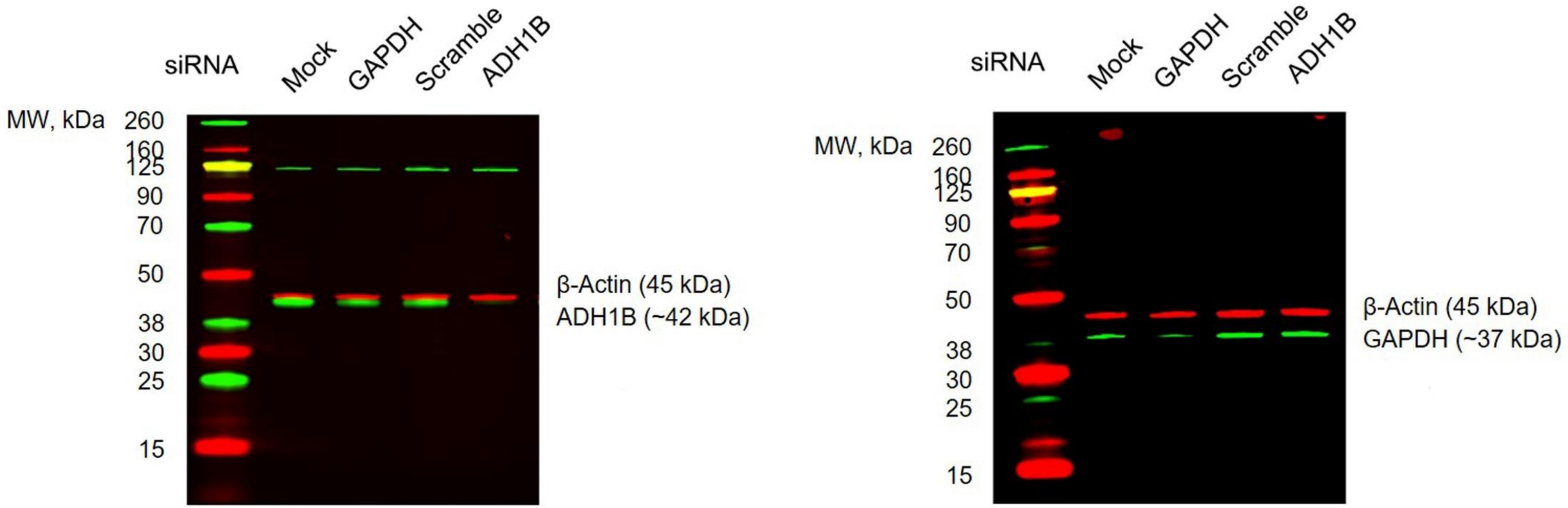

Supplementary Figure S12. Knockdown of ADH1B expression via siRNA. Two-color fluorescent immunoblot analysis of cell lysates following transfection with siRNA. Pre-adipocytes derived from lean (BMI 21.6 kg.m-2) donor tissue were differentiated to adipocytes. At Day 9-10 during differentiation, cells were transfected with specific pooled siRNA targeting *ADH1B* or *GAPDH* (positive control) gene. Cells transfected with non-targeting (Scramble) siRNA or vehicle alone (Mock) were utilized as negative controls. Total protein was isolated, resolved by SDS-PAGE, and immunoblotted with antibodies specific for ADH1B (800 nm channel), GAPDH (800 nm channel) and β-Actin (700 nm channel) loading control. Samples were derived from the same experiment and processed in parallel. Fluorescence intensity was measured using the LI-COR Odyssey CLx infrared imaging system (Resolution: 169µm; Intensity: auto; Quality: lowest). Positive control confirmed the efficacy of siRNA-targeted knockdown and negative controls confirmed that siRNA knockdown by transfection is specific and nontoxic.

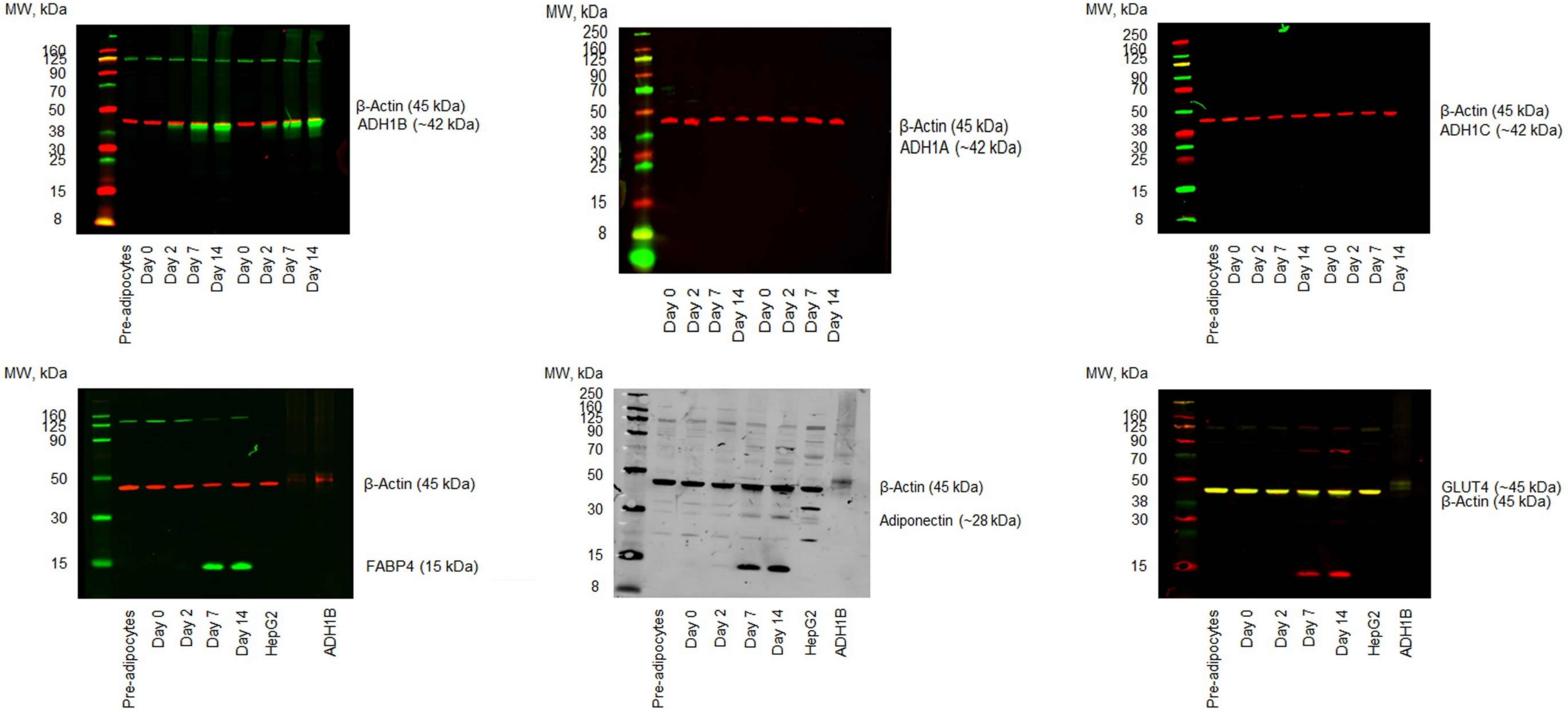

Supplementary Figure S13. Expression levels of ADH1 and other adipocyte proteins during adipogenesis. Representative images of two-colored fluorescent immunoblot analysis of cell lysates from human subcutaneous pre-adipocytes (Day 0) derived from lean (BMI < 30 kg.m^{-2}) donor tissue during differentiation to cultured mature adipocytes (Day 14). Total protein was collected at the indicated time points during differentiation, resolved by SDS-PAGE, and immunoblotted with antibodies specific for the three ADH1 isoforms: ADH1A (800 nm channel), ADH1B (800 nm channel), ADH1C (800 nm channel); the adipokines FABP4 and adiponectin; and GLUT4. β-Actin (700 nm channel) was used as a loading control. Samples were derived from the same experiment were processed in parallel. Fluorescence intensity was measured using the LI-COR Odyssey CLx infrared imaging system (Resolution: 169µm; Intensity: auto; Quality: lowest). LI-COR iS Image Studio software was used for image modification (channel realignment, brightness, contrast, color inversion) and data analysis.

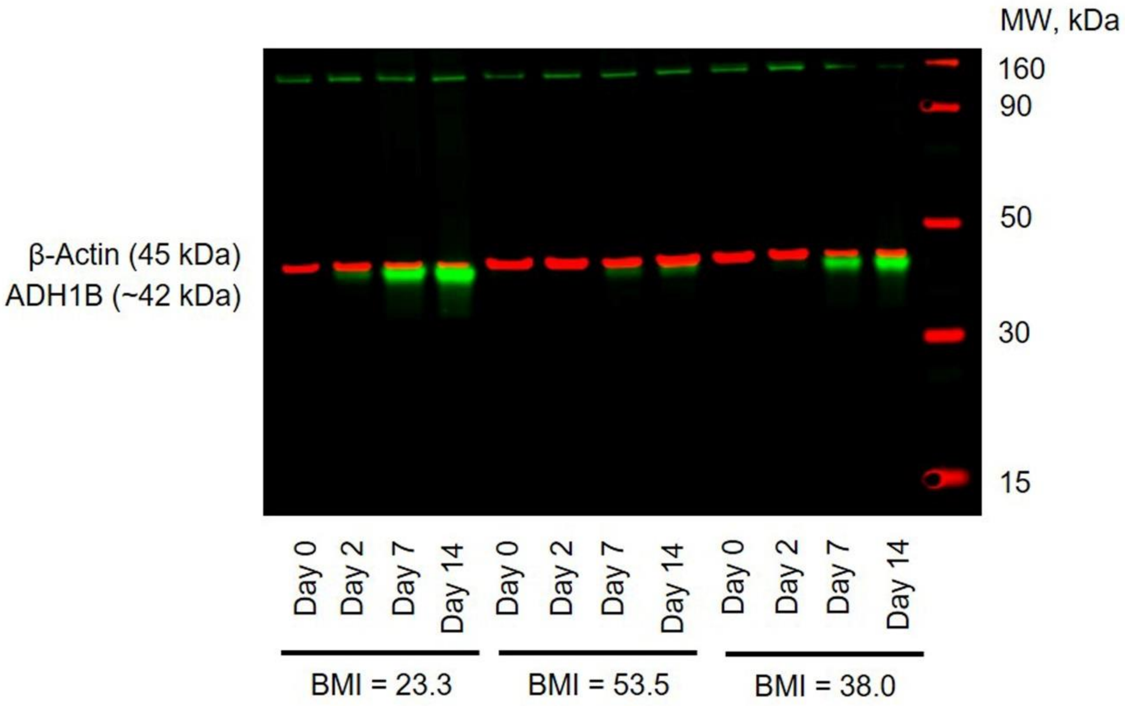

Supplementary Figure S14. ADH1B protein expression in adipocytes decreased with increasing BMI. Representative image of two-color fluorescent immunoblot analysis of cell lysates from lean (BMI < 30 $kg.m^{-2}$) and obese (BMI ≥ 30 $kg.m^{-2}$) adipocytes during adipogenesis. Total protein was collected at the indicated time points during differentiation, resolved by SDS-PAGE, and immunoblotted with antibodies specific for ADH1B (800 channel) and β-Actin (700 channel) loading control. Fluorescence intensity was measured using the LI-COR Odyssey CLx infrared imaging system (Resolution: 169µm; Intensity: auto; Quality: lowest).

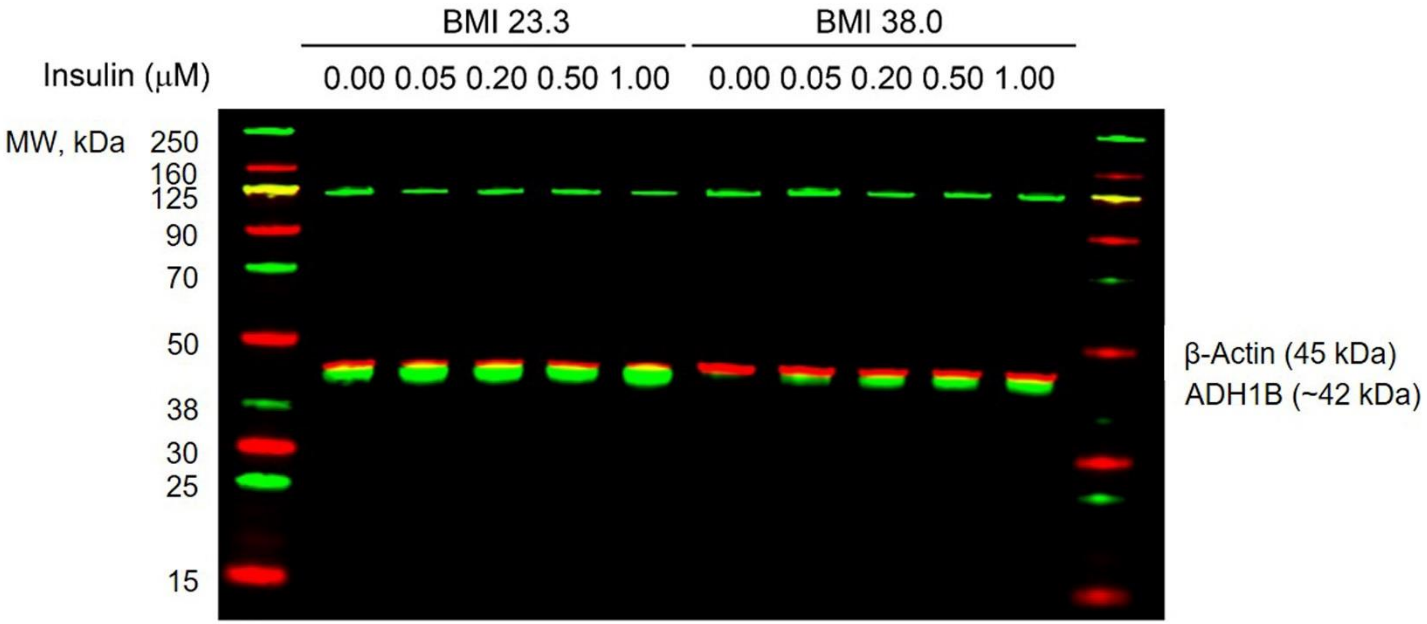

Supplementary Figure S15. Insulin promotes expression of ADH1B in lean and obese adipocytes. Representative image of two-color fluorescent immunoblot analysis of cell lysates from lean (BMI < 30 kg.m^{-2}) or obese (BMI ≥ 30 kg.m^{-2}) adipocytes following treatment with increasing doses of insulin. Cells were starved for 12 h and then treated with the indicated dose of insulin for 1 h. Total protein was isolated, resolved by SDS-PAGE, and immunoblotted with antibodies specific for ADH1B (800 nm channel) and β-Actin (700 nm channel) loading control. Fluorescence intensity was measured using the LI-COR Odyssey CLx infrared imaging system (Resolution: 169µm; Intensity: auto; Quality: lowest).

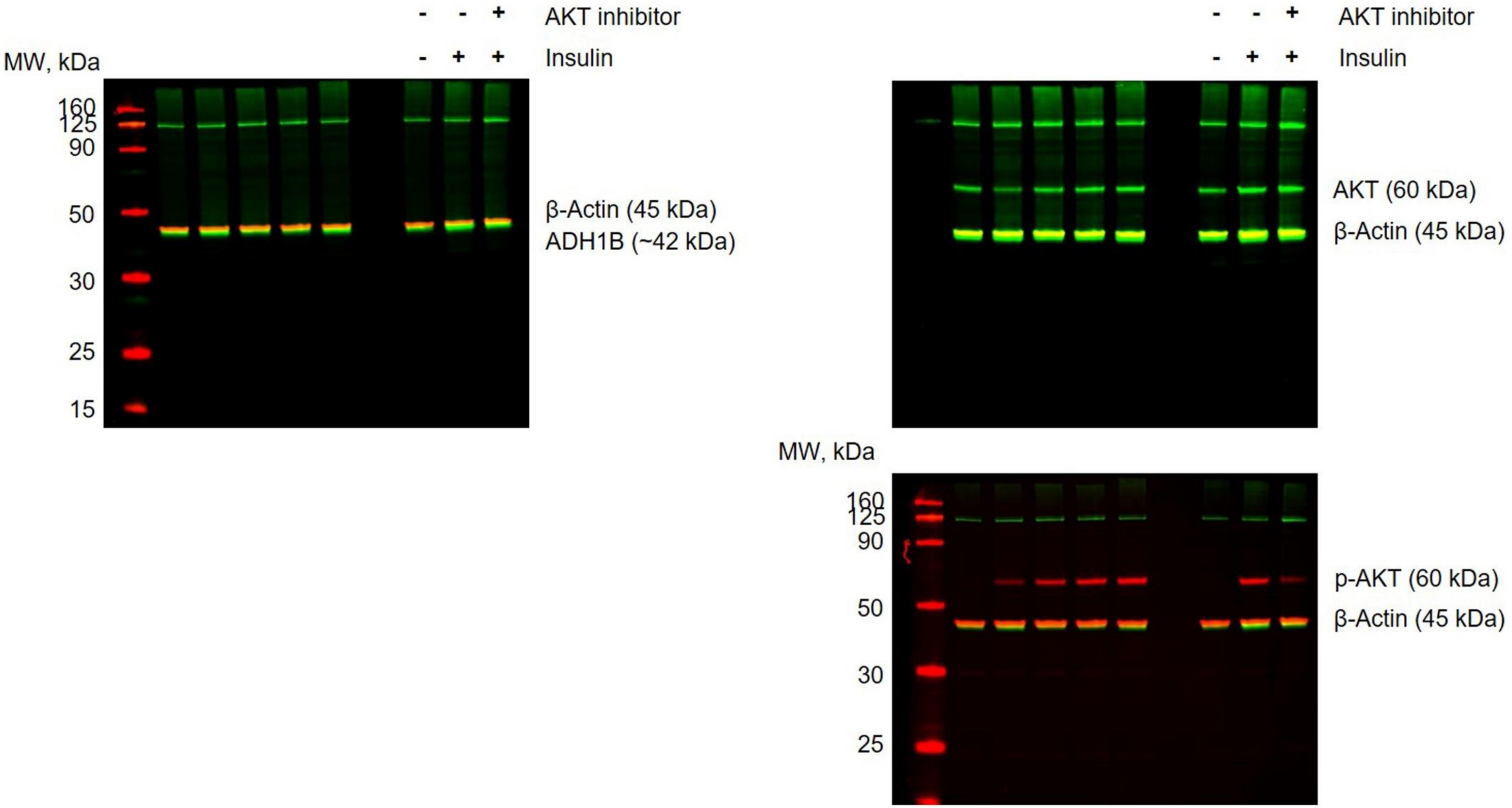

Supplementary Figure S16. AKT is involved in insulin-mediated ADH1B expression**.** Representative image of two-colored fluorescent immunoblot analysis of cell lysates from lean (BMI < 30 kg.m-2) adipocytes with (+) and without (-) treatment with AKT inhibitor and/or insulin. Cultured adipocytes were starved for 12 h and then treated with 20 µM AKT1/2-specific inhibitor (+AKT inhibitor) for 1 h prior to treatment with 0.05 µM insulin (+Insulin) for 1 h. Untreated cells (-insulin, -AKT inhibitor) were utilized as controls. Total protein was isolated, resolved by SDS-PAGE, and immunoblotted with antibodies specific for phosphorylated AKT (p-AKT, 700 nm channel), total AKT (800 nm channel) and ADH1B (800 nm channel). β-Actin (700 nm channel) was used as loading control. Samples derived from the same experiment were processed in parallel. Fluorescence intensity was measured using the LI-COR Odyssey CLx infrared imaging system (Resolution: 169µm; Intensity: auto; Quality: lowest).
